# Lifetime healthcare expenditures across socioeconomic groups

**DOI:** 10.1186/s12889-024-20209-1

**Published:** 2024-10-09

**Authors:** Malene Kallestrup-Lamb, Alexander O. K. Marin

**Affiliations:** 1 Aarhus University and PeRCent, Fuglesangs Allé 4, 8210 Aarhus, Denmark; 2 Present Address: University of Southern Denmark, Campusvej 55, 5230 Odense, Denmark; 3 Aarhus University, Aarhus, Denmark

**Keywords:** Lifetime healthcare expenditures, Socioeconomic groups, Inequality, Social gradient, Mortality, I14, H51

## Abstract

**Background:**

A socioeconomic gradient affects healthcare expenditures and longevity in opposite directions as less affluent individuals have higher current healthcare expenditures but simultaneously enjoy shorter lives. Yet, it is unclear whether this cross-sectional healthcare expenditure gradient persists from a lifetime perspective. This paper analyzes lifetime healthcare expenditures across socioeconomic groups using detailed individual-level healthcare expenditure data for the entire Danish population.

**Method:**

Using full population healthcare expenditures from Danish registries, we estimate lifetime healthcare expenditures as age-specific mean healthcare expenditures times the probability of being alive at each age. Our data enables the estimation of lifetime healthcare expenditures by sex, socioeconomic status, and by various types of healthcare expenditure.

**Results:**

Once we account for mortality differences and all types of healthcare expenditures, all socioeconomic groups spend an almost equal amount on healthcare throughout a lifetime. Lower socioeconomic groups incur the lowest lifetime hospital expenditures, whereas higher socioeconomic groups experience the highest lifetime expenditures on long-term care services. Our findings remain robust across various socioeconomic measures and alternative estimation methodologies.

**Conclusion:**

Improving the health status of lower socioeconomic groups to align with that of higher socioeconomic groups is costly but may ultimately reduce current healthcare expenditures. Enhanced health outcomes likely increase lifespan, leading to extended periods of healthcare consumption. However, since all socioeconomic groups tend to have similar lifetime healthcare expenditures, this prolonged consumption has limited impact on overall lifetime healthcare costs. Additionally, a significant benefit is the deferment of healthcare expenditures into the future. Overall, our results diminish concerns about socially inequitable utilization of healthcare resources while socioeconomic differences in health and longevity persist, even in a universal healthcare system.

**Supplementary Information:**

The online version contains supplementary material available at 10.1186/s12889-024-20209-1.

## Introduction

National healthcare expenditures are increasing across the globe [77, 93] due to aging populations [57, 58], costly health technologies [13], prescription drug expenditures [47, 80], and end-of-life healthcare expenditures [30, 39]. This development challenges the sustainability of healthcare systems and thus leads to an increased focus on cost management and socioeconomic differences in healthcare expenditures. Moreover, it raises questions concerning the social equability of healthcare utilization [4] and its benefits [17] within free healthcare systems. Researchers have found that lower socioeconomic groups generally have higher healthcare expenditures compared to higher socioeconomic groups [19, 25], typically referred to as a negative social gradient in healthcare expenditures [38]. In Denmark, for example, the poorest 20% above age 65 spend more than twice as much on healthcare as the richest 20% [16].

Studies of the negative social gradient in healthcare expenditures typically compare expenditures between socioeconomic groups within a single year and do not account for mortality differences between groups. However, lower socioeconomic groups face larger risks of mortality [12, 66, 87], have poorer health [11, 51, 72], and are additionally at a disadvantage as they have shorter life expectancies [14, 55], labeled the social gradient in mortality [60]. Consequently, lower socioeconomic groups consume relatively higher healthcare expenditures across a shorter lifetime, whereas higher socioeconomic groups spend less on healthcare but on average live longer. Eliminating the socioeconomic gradient in healthcare expenditures and mortality is associated with longer lifespans and potentially better overall health outcomes, both of which are generally considered desirable. A critical question, however, is whether the additional years of life in which healthcare expenditures are incurred exceed the lower annual expenditures used in each year alive. In particular, the adversely related healthcare expenditure and longevity gradients make it unclear whether a lifetime healthcare expenditure gradient exists once we account for socioeconomic longevity difference. It is essential to consider whether eliminating the socioeconomic gradients would ultimately increase or decrease the total healthcare expenditures across the lifespan of an individual to quantify the financial gains or losses associated with reducing the gradients.

Lifetime healthcare expenditures have been estimated using a variety of methods [10, 34, 37, 56, 64], but only a few of these studies investigated the inequality in lifetime healthcare expenditures [26, 48, 96, 97]. Of particular interest are Asaria et al. [5], who pioneered classifying individuals into socioeconomic groups and estimating lifetime healthcare expenditures. Using English data, they find a 10% negative gradient in lifetime inpatient hospital expenditures for both men and women. However, their analysis focuses solely on hospital expenditures and thereby omits many sizable types of healthcare expenditures, such as expenditures for outpatient hospital care and long-term care.

This paper presents an important contribution in the investigation of the negative social gradient in lifetime healthcare expenditures. The Danish healthcare system offers a uniquely advantageous setting for analysis of socioeconomic differences in healthcare expenditures since residents have equal access, and healthcare is free of charge and paid for by the universal healthcare insurance [62]. We estimate lifetime healthcare expenditure across gender and socioeconomic groups following the easily interpretable methodologies from Asaria et al. [5]. The methodology weights the average healthcare expenditures accumulated throughout a lifetime by a representative individual with the percentage of individuals that die at each age. Unlike previous studies, our research examines a complete set of healthcare expenditure components, including inpatient and outpatient hospital care, prescription drugs, primary care physicians, nursing homes, home care, and home nurses. By using population-wide Danish register data, we are able to extend the top-coded age and capture socioeconomic discrepancies in mortality and healthcare consumption at extreme ages, which is a second significant contribution. Finally, we propose to test the statistical significance of lifetime healthcare expenditure differences, which has not been done before.

Analyses of the average annual healthcare expenditures confirm the canonical result that lower socioeconomic groups tend to spend the most on all types of healthcare expenditures - in particular, from age 30 to around age 80. Yet, by leveraging the detail of our data and raising the top-coded age to 100, an intriguing positive social gradient for primary care physician services, inpatient hospital care, and outpatient hospital care emerges between age 80 and 100+. Specifically, the highest socioeconomic group annually consumes more of these types of expenditure types than the lower socioeconomic groups. We caution, however, that the point estimates display a large disparity as the use of expenditures at extreme ages fluctuate.

Our initial estimates of total lifetime healthcare expenditures ignore, momentarily, any socioeconomic mortality differences. A differential level of healthcare expenditures is used across the socioeconomic groups while all males and females, respectively, face equal mortality exposure. We find large socioeconomic differences in lifetime healthcare expenditures, i.e. 54.6% for males and 32.1% for females. These estimates simply extrapolate the usual negative social gradient in average annual healthcare expenditures to the lifetime perspective. Meanwhile, socioeconomic longevity differences are repeatedly confirmed in demographic research [14, 55], presenting an important dimension to the consumption of healthcare expenditures across a lifetime. Accounting for socioeconomic-specific levels of mortality reduces the gradient in total lifetime healthcare expenditures by more than 32 percentage points. The difference is now 8% for women and five for men. The lower socioeconomic groups simply consume more healthcare expenditures each year alive but enjoy shorter lives on average. Despite being significantly reduced, a Welch [89] test revealed that the differences remain statistically significant and the lowest socioeconomic groups have the highest expected total lifetime healthcare expenditures from age 30 to death. Moreover, we find that lifetime healthcare expenditures for women are one-third larger than for men due to longer lifespans for women and gender differences in annual age-specific healthcare expenditures.

Estimating lifetime expenditures by different types of healthcare expenditures, we confirm socioeconomic differences in lifetime inpatient hospital care as in Asaria et al. [5]. Yet, as higher socioeconomic groups generally enjoy longer lifespans, it enables them to spend more on home care, home nurses, and nursing homes throughout their lifetime. Specifically, we document a novel positive socioeconomic gradient in lifetime nursing home expenditures as well as lifetime home care expenditures where the highest socioeconomic group spends the most in a lifetime. As such, the positive gradient in long-term care counteracts the negative gradient in inpatient hospital expenditures, which has an equalizing effect on total lifetime healthcare expenditures.

Our main results are robust across measures of socioeconomic status and different measures of average annual healthcare expenditures. Yet, top-coding the age dimension proves highly impactful on the socioeconomic differences in lifetime healthcare expenditures. Lowering the top-coded age to 85, which often occurs in studies with limited data, re-inflates the socioeconomic difference in lifetime healthcare expenditures by more than 33 percentage points, i.e. almost equal to the overlooking socioeconomic mortality difference. This finding signifies the stark socioeconomic differences present beyond age 85. The highest socioeconomic groups both face the lowest mortality risks and have the highest average healthcare expenditure usage on many sizable expenditure components.

We stress that several different benefits should be considered in analysis of health improvements targeted at lower socioeconomic groups. Better health is beneficial for the individual, including marginal improvements in health each year as well as through a longer lifespan [9, 20]. However, raising the latent health status, affecting mortality and healthcare consumption of individuals in lower socioeconomic groups to match that of higher socioeconomic groups, could be costly. Our results suggest that such health improvements have the potential to reduce total lifetime expenditures. As health status affects healthcare expenditures [24], improving health can bring the average annual healthcare expenditures of lower socioeconomic groups closer to those of higher socioeconomic groups, thereby decreasing total lifetime healthcare expenditures. Moreover, as expenditures will be spread over a longer life, the financial burden of healthcare expenditures will shift into the future, which may have significant policy-relevant implications for healthcare systems.

The remainder of the paper proceeds as follows. “Data” section describes the Danish healthcare system, the data used for estimation, and the socioeconomic measure. The estimation method is described in “Method” and “Results” sections reports our results. “Discussion” section provides a discussion of our findings and hereafter “Conclusion” section concludes the paper.

## Data

### The danish healthcare system

The Danish healthcare system can be characterized as a Beveridgian model [6]. Citizenship enables free and equal access to universal health insurance, including high-quality services such as in- and outpatient somatic and psychiatric care, emergency care, primary care practitioners, specialist practitioners, home care, home nurses, nursing homes for the oldest, and high subsidies for prescription drugs [62]. The responsibility for healthcare is organized at three levels. The state has the overall regulatory and supervisory responsibility for healthcare and long-term care. The five administrative regions in Denmark oversee the primary care physicians, other medical specialists, and all types of hospital care. The responsibilities of the 98 municipalities include home help, home nurses, and nursing homes [62].

Taxes primarily finance health expenditures, and in 2012 Denmark spent 10.2% of its GDP on healthcare [91]. This is higher than the OECD average of 8.9% [62] but lower than the 17% in the United States [69]. To manage expenditures, primary care practitioners serve as gatekeepers, and patients need a referral to access hospital and specialist treatment [71]. Moreover, prescription drugs, dental care, physiotherapist, and psychological services are subject to limited out-of-pocket payments [71], which amounted to only 14% of all healthcare expenditures in Denmark. This is lower than the EU average of 16% [67]. To insure against such statutory co-payments, 40% of Danes held a complementary health insurance in 2012 [23]. Despite the relatively high insurance coverage, private voluntary health insurance costs only accounted for about 3% of all healthcare expenditures [68, 92].

### Danish registry data

Our dataset consists of detailed population-wide administrative registers for the year 2012, combining data from Statistics Denmark and the Danish Health Data Authority using anonymized keys. Statistics Denmark provides yearly information on gender, income, wealth, year of death, and age. We consider all individuals above the age of 30, which allows us to obtain a well-defined socioeconomic measure. Moreover, the registers enable investigation of differences in socioeconomic expenditures at extreme ages, i.e., requiring only top-coding from age 100. As only a single year of data is available, we utilize the entire age dimension to capture the life cycle of healthcare expenditures.

Health information is retrieved from four different registers. A unique feature of our study is our access to daily expenditure information at the individual level on hospital admission, visits to the primary healthcare sector, all purchases of prescription drugs, and long-term care. Combining all types of healthcare expenditures was only possible in the year 2012 due to data restrictions. However, in Denmark, healthcare expenditures have consistently represented a constant share of the GDP since 2010, except during the Covid pandemic [68], reflecting a stable resource allocation. Thus, the results are believed to be representative for more recent years. First, the Diagnosis-Related Group (DRG) National Patient Registry provides daily expenditure data on individual in- and outpatient treatments in all Danish hospitals for somatic and psychiatric patients. The DRG registry provides unit costs by diagnosis and activities for individual hospital services, which include some fixed costs [79, 85]. Second, the National Health Insurance Service Registry contains the reimbursed amount for all services provided to patients by primary care physicians, practicing medical specialists, physiotherapists, psychologists, and chiropractors [3], covered by the universal health insurance. Data is recorded on a weekly basis. Third, the Register of Medicinal Product Statistics provides daily information on the reimbursed amount of all prescription drugs sold to individuals [54], which accounts for around 80% of turnovers in pharmacies [16]. Drugs sold to hospitals are included in the DRG expenditures.

Fourth, Statistics Denmark provides daily data on the utilization of long-term care, which includes nursing home residency for the most fragile, while those who can take care of themselves with limited assistance live in residential houses or their own homes. The registers contain information on the date an individual moves into and out of a nursing home or residential home. Home carers provide personal care such as bathing, administering medication, getting dressed, and getting out of bed, as well as practical help, e.g. cleaning and food preparation. As practical help is not directly health-related, we exclude these services. The purpose of home nursing is to prevent disease, promote health, provide nursing care and treatment, rehabilitation, and palliation to patients who need it.

To quantify individual expenditure to each type of long-term care, we follow the approach in Christensen et al. [16] and distribute national expenditures to home carers and home nurses, respectively, according to minutes serviced to the individual. Similarly, national nursing home expenditures are distributed by residential status. An accurate national amount for the services we consider is unavailable in 2012. Instead, we use the 2008 total public expenditures on long-term care from the Danish Economic Council [22] and project it to the 2012 level according to the corresponding growth rates in the OECD [68] Data.^1^

Our dataset encompasses comprehensive healthcare expenditures, covering inpatient and outpatient hospital care, prescription drugs, primary care, and long-term care services. Notably, long-term care is not universally classified as part of healthcare across all systems. In alignment with the System of Health Accounts [90], this paper includes long-term care services related to personal care, home nursing, and nursing homes within the healthcare expenditure category while excluding non-medical home care activities, such as shopping and cleaning.

Appendix A.1, Table A.1 provides summary statistics of our data. While women have the highest average total expenditures, males use the most inpatient hospital expenditures on average. Moreover, males tend to have higher average levels of both income and wealth.

### Socioeconomic measures

To partition individuals into socioeconomic groups, we use the affluence measure by Cairns et al. [8], which is especially suitable for our dataset [52] as it measures socioeconomic status at the individual level. Affluence includes both income and wealth, which provides a more complete picture of an individual’s economic status. Wealth in particular reflects accumulated assets such as property, investments, savings, and inherited wealth, which can significantly influence an individual’s socioeconomic status. Including both is important as they are equally likely to proxy adherence to a socioeconomic group, i.e. an individual can have either high income, high wealth, or both. Further, as income and wealth correlate with healthcare expenditures separately [32], considering both are essential for our analysis of socioeconomic differences in healthcare expenditures. The affluence measure, $A_{i,x,t}$, for individual, *i*, aged, *x*, in year, *t*, combines *K* times lagged income, $I_{i,x-1,t-1}$, with lagged wealth, $W_{i,x-1,t-1}$, for every individual in the dataset

1 $$\begin{aligned} A_{i,x,t} = K \cdot I_{i,x-1,t-1} + W_{i,x-1,t-1} \,. \end{aligned}$$

As income and wealth might be missing in the year of death, using lagged values of income and wealth is advantageous. *K* serves as a capitalization factor to approximate the present value of future retirement income. Fixing K across all ages could oversimplify the affluence measure and potentially distort the balance between income and wealth, particularly as life expectancy fluctuates across different age groups. Adjusting K based on life expectancy would ensure a more precise measure of affluence that adjusts appropriately for the expected duration over which retirement income must be spread. However, the potential gains in precision might be marginal, given that the affluence measure is already robust to a range of K values (between 10 and 20 [8]). Thus, the potential improvement in the accuracy of the affluence measure may be limited, especially when considering the added complexity. We follow Cairns et al. [8] and set *K* equal to 15.

We use a gender-specific allocation procedure to assign each individual in each year and age into one of five equally-sized socioeconomic groups based on the individual’s ranking in the affluence measure. Thus, by each age, year, and gender, the 20% least affluent enter socioeconomic group 1 (SEG1), the second-lowest 20% enter the second-lowest group (SEG2), and so on until the 20% most affluent enter socioeconomic group 5 (SEG5). Allocating individuals consistently by age, year, and gender ensures a balanced socioeconomic representation across age groups and prevents the overrepresentation of wealthier, higher-income age groups in the top socioeconomic categories. Meanwhile, at retirement, there is limited variation in income; thus, we fix the socioeconomic group in that year as suggested by Cairns et al. [8].^2^ In contrast to this socioeconomic measure, Asaria et al. [5] use a deprivation index that assigns all individuals in a large neighborhood of an average size of 1,500 to the same socioeconomic group.

We recognize that other measures of socioeconomic status, such as education, occupational status, income, wealth, and combinations thereof, have been suggested [33, 35, 42, 55, 59, 83] and perform a robustness check of our results using educational attainment as a socioeconomic measure. Appendix A.3 defines educational measures. Early-life health events with onset before age 30 will likely affect adherence to a socioeconomic group by constraining educational attainment, limiting job prospects, or determining the type of employment an individual can pursue. Hvidberg et al. [49] indeed find that the prevalence of mental health disorders and neurological diseases is significantly higher among younger individuals with lower levels of education. However, they also find that socioeconomic disparities in all other diseases are less pronounced.

From the summary statistics in Appendix A.1, Table A.1, we note that lower socioeconomic groups spend more on each type of healthcare expenditure on average, as found in existing literature [16, 19]. For males, the lowest group spends $5,878 on average and $3,989 for the highest socioeconomic group. The average spending is higher for females, i.e., $6,739 and $5,340, respectively.

## Method

### Lifetime healthcare expenditure estimates

This section describes our method to estimate lifetime healthcare expenditure, which follows Asaria et al. [5] closely. Before providing the formula for lifetime healthcare expenditures, we define its different components.

We calculate the average healthcare expenditures, $\overline{\text {HCE}}_{x,s,g,t}$, for year, *t*, in Eq. (2) by gender, *g*, socioeconomic group, *s*, and cost component at each age, *x*, as the sum of individual healthcare expenditures, *hce*, used by all individuals, $i=1,\dots ,N_{x,s,g,t}$, divided by the number of individuals:

2 $$\begin{aligned} \overline{\text {HCE}}_{x,s,g,t} = \frac{ \sum _{i=1}^{N_{x,s,g,t}} hce_{i,x,s,g} }{N_{x,s,g,t}} \,. \end{aligned}$$

In a similar fashion, Eq. (3) calculates mortality rates as the number of deaths divided by the number of individuals who are at risk of dying. The binary indicator, *die*, takes the value one if an individual dies and zero otherwise:

3 $$\begin{aligned} D_{x,s,g,t} = \frac{\sum _{i=1}^{N_{x,s,g,t}} die_{i,x,s,g}}{N_{x,s,g,t}} \,. \end{aligned}$$

Turning to the lifetime perspective, a representative individual use the average healthcare expenditures in each year alive from the minimum age, $x_{min}$, to death, *d*. We define the cumulative healthcare expenditures, $\text {HCE}^{cum}_{d,s,g,t}$, spent across a lifetime conditional on the age of death as

4 $$\begin{aligned} \text {HCE}^{cum}_{d,s,g,t} = \sum \limits _{x=x_{min}}^{d} \overline{\text {HCE}}_{x,s,g,t} \,, \end{aligned}$$

which accumulates the average healthcare expenditures observed in cross-sectional data across the age dimension.

The amount $\text {HCE}^{cum}_{d,s,g,t}$ varies by age of death, gender, and socioeconomic group. The percentage of individuals that die at age, *d*, survive all ages from $x_{min}$ to age $d-1$ and is calculated as

5 $$\begin{aligned} \text {mort}_{d,s,g,t} & = P\left( d \mid \text {survive ages } x_{min} \text { to } d-1 \right) \nonumber \\ & = D_{d,s,g,t} \prod _{x=x_{min}}^{d-1} \left( 1-D_{x,s,g,t}\right) \,. \end{aligned}$$

At the minimum age, we set $\text {mort}_{x_{min},s,g,t}$ to $D_{x_{min},s,g,t}$ since we assume that everyone is alive prior to the minimum age. At the maximum age, $x_{max}$, we superimpose that no one outlives the maximum age and set $\text {mort}_{x_{max},s,g,t}$ to $1-\sum _{x=x_{min}}^{x_{max}-1} \text {mort}_{x,s,g,t}$.

Mean lifetime healthcare expenditures, $\text {LHE}_{s,g,t}$, are estimated by gender and socioeconomic group using a similar method as Asaria et al. [5]. We weight cumulative healthcare expenditures from Eq. (4) by the percentage of individuals that die at each age calculated in Eq. (5)

6 $$\begin{aligned} \text {LHE}_{s,g,t} = \sum \limits _{x=x_{min}}^{x_{max}} \text {mort}_{x,s,g,t} \cdot \text {HCE}^{cum}_{x,s,g,t} \,. \end{aligned}$$

The estimate of lifetime healthcare expenditures in Eq. (6) is asymptotically equivalent to the simulation based approach first applied to estimate lifetime healthcare expenditures in Alemayehu and Warner [2]. Instead of simulating stochastic healthcare expenditures and mortality paths for many individuals, we simply calculate the mean of lifetime expenditures using discrete expectations, which is feasible in large datasets. The Asaria et al. [5] method has the advantage of not requiring distributional assumptions and having low computational costs. The Asaria et al. [5] approach used in this paper can be considered a period-lifetable approach as it uses data from a single year, *t*. This approach results in an artificial population with age, sex, and socioeconomic group-specific levels of mortality and mean healthcare expenditures calculated at the year, *t*, level. Rather than using the probability of dying, $\text {mort}_{x,s,g,t}$, the observed mortality rate, $D_{x,s,g,t}$, could be considered in its place in the calculation of lifetime healthcare expenditures in Eq. (6). This can be regarded a *cross-sectional* approach. Doing so reflects the mortality patterns of the current population and its size without considering an artificial population. A drawback of the cross-sectional approach, however, is that it may either overestimate or underestimate the number of deaths, as the number of deaths is not guaranteed to sum to unity, unlike the period-lifetable approach. If the sum of mortality rates, $D_{x,s,g,t}$, is less than one, not all individuals in the population die, which has a decreasing effect on lifetime healthcare expenditures, while the reverse is true when the sum exceeds one. This debilitates the interpretations of our outcome of interest being the expected lifetime healthcare expenditures for a socioeconomic group. Alternatively, a cohort life-table approach could be considered, which would involve tracking the mortality rates and healthcare expenditures of a specific cohort over time, such as those born a century ago. Yet, considering the immense changes over this period with respect to medical practice, technology, and living standards, the relevance of such historical estimates to current conditions may be limited. Moreover, it is infeasible to pursue the cohort-lifetable approach since historical data on healthcare expenditures across several decades is unavailable. We continue our reflections on the Asaria et al. [5] estimation approach in “Discussion” section.

### Statistical tests of differences in lifetime healthcare expenditure

Comparing mean lifetime healthcare expenditures in Eq. (6) across socioeconomic groups uncovers whether differences are quantitatively important. Yet, to assess if these differences arise from sampling errors, rigorous statistical tests are needed. Unlike the existing literature, we suggest testing whether socioeconomic groups use the same mean lifetime healthcare expenditures by applying the two-sided Welch t-test of equal means [89]. Compared to the Student’s t-test [84], this test has the attractive feature that it allows for tests of means estimated from separate samples. This is particularly relevant when using data from distinct socioeconomic groups.^3^ We set up the standard Welch test in Eq. (7), which divides the differences in mean lifetime healthcare expenditures in socioeconomic groups $s_1$ and $s_2$ by the square root of its variance

7 $$\begin{aligned} t_W = \frac{\text {LHE}_{s_1,g,t} - \text {LHE}_{s_2,g,t}}{\sqrt{ {Var\left( \text {LHE}_{s_1,g,t}\right) } + {Var \left( \text {LHE}_{s_2,g,t}\right) } }} \,. \end{aligned}$$

As mean lifetime healthcare expenditures are estimated using separate samples, the covariance between mean lifetime healthcare expenditures in socioeconomic groups $s_1$ and $s_2$ is assumed to be zero and hence does not appear in the denominator of Eq. (7). This key feature sets the Welch test apart from other tests. Evidently, from Eq. (6), calculating mean lifetime healthcare expenditures involves the sum of products where each constituent in itself is either a sum or product of other terms. Consequently, deriving the variance of lifetime healthcare expenditures, $Var\left( \text {LHE}_{s,g,t}\right)$, is complicated and involves non-standard variance and covariance calculation rules. Thus, we resolve to bootstrapping standard errors [29] for simplicity. Specifically, we re-estimate lifetime healthcare expenditures by conducting a bootstrap resampling of the individual data within each gender-specific socioeconomic group, repeating this process 1,000 times as detailed in “Lifetime Healthcare Expenditure Estimates” section. The resampling is performed with replacement and maintains a sample size equal to that of the original group as is best practice [46]. The usefulness of testing lifetime differences applies to other research questions, e.g. whether smoking significantly reduces lifetime medical expenditures [98], which are typically evaluated by comparing numerical values.

## Results

### Average and cumulative healthcare expenditures

**Fig. 1:**
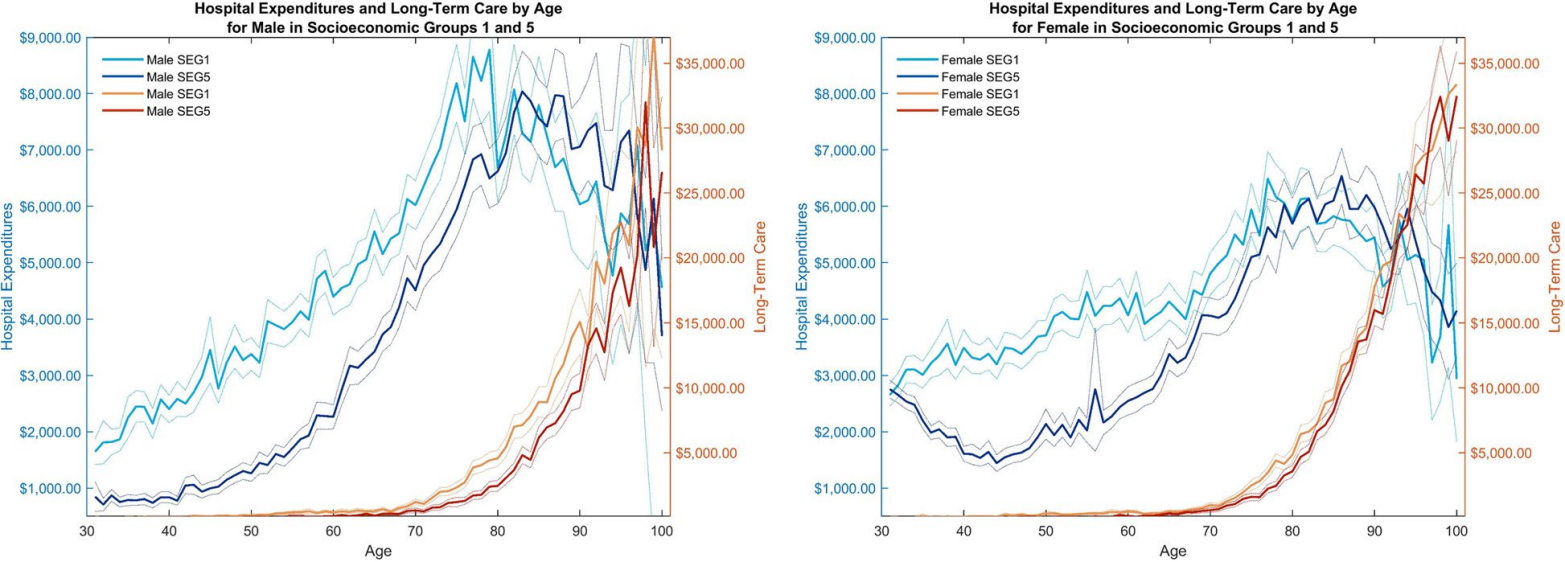
Average hospital (Blue) and long-term care (Orange) expenditures by age, gender, and socioeconomic group. Dotted outline of 95% confidence bands. Amounts are in 2012 USD

**Fig. 2:**
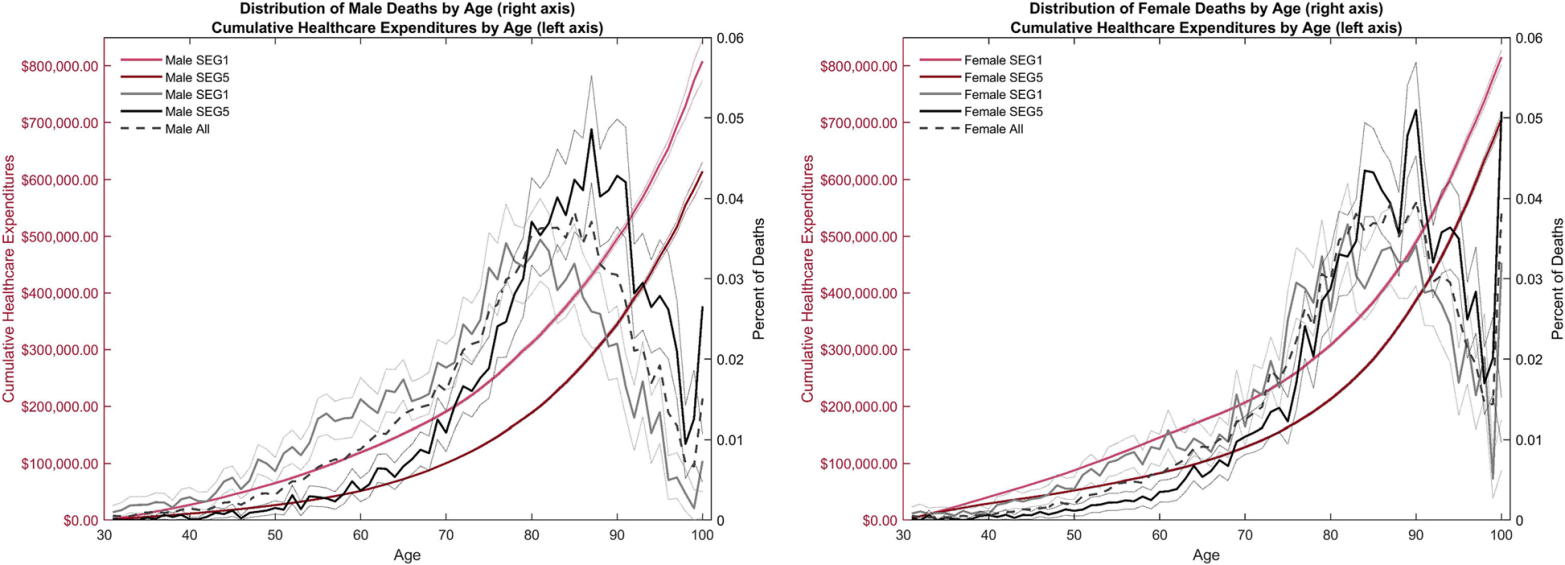
Percentage of deaths (Grey/Black) and cumulative healthcare expenditures (Red) by age and socioeconomic group. Dotted outline of 95% confidence bands. Amounts are in 2012 USD

First, we analyze the largest annual healthcare component, in- and outpatient hospital expenditures, in the age-dimension for males (left) and females (right) in Fig. 1. We account for socioeconomic differences using the average of healthcare expenditures in Eq. (2). The average increases with age for both genders and peaks around age 75 for the lowest socioeconomic group (SEG1, light blue) and ten years later for the highest socioeconomic group (SEG5, dark blue). Hereafter, hospital expenditures decline. We confirm the negative social gradient from age 30 to 80, where the lowest socioeconomic group spends the most in hospitals. With the detail of our data we, unlike existing literature, document a positive gradient in average hospital expenditures from age 80 to 100+ where the most affluent group has the highest average expenditures. The inversion of the negative social gradient in hospital expenditures likely arises from a survivorship bias as only the healthiest individuals in the lowest socioeconomic group survive into older ages. While survivorship bias is present across all socioeconomic groups, new evidence indicates that individuals in lower socioeconomic groups face a higher likelihood of transitioning to poorer health and a reduced probability of recovery compared to their more affluent counterparts [53]. Furthermore, this inversion may reflect socioeconomic disparities in the age distribution of death, with the modal age of death occurring after age 80 in higher socioeconomic groups as compared to lower socioeconomic groups where the modal age at death is earlier (Fig. 2). This difference in age at death likely contributes to higher average hospital expenditures, as end-of-life care is strongly associated with significant hospital costs [39]. Finally, a substitution between hospital and elderly care may vary across socioeconomic groups at older ages. Large variations in hospital expenditures between ages 80 and 100+, nonetheless, makes the 95% confidence bands overlap.

Despite equal access to hospital care, we observe a clear gender difference in hospital expenditures. As commonly found [31, 88], males generally have higher hospital expenditures than females, in particular at older ages. Females in higher socioeconomic groups generally have their first child after the age of 30, which occurs at a later stage compared to their counterparts in lower socioeconomic groups [63]. This delay in childbearing partially accounts for the widening disparity in hospital expenditures observed between the ages of 30 and 40, as females in the highest socioeconomic group incur higher maternity costs during their thirties.^4^ Given that total fertility rates were similar across all socioeconomic groups in 2012 [50], maternity expenditures will only modestly contribute to widening the gap. Compared to Christensen et al. [16], who use Danish register data to calculate average annual healthcare expenditures for individuals above and below age 65, respectively, our hospital expenditure estimates are comparable. Individuals below age 65 spend on average $2,553 annually, while individuals above age 65 consume $5,416 in hospital expenditures annually.

The second largest cost component, long-term care, is represented by the orange lines on the right axis of Fig. 1. It is defined as the sum of average healthcare expenditures to home care, home nurses, and nursing homes. From ages 30 to 70, average long-term care expenditures are close to zero as only a small part of the population receives long-term care. After age 70, average expenditures steadily increase to about $27,500 at age 100+. The lowest socioeconomic groups (light orange) generally have higher long-term care expenditures from age 70 to 100+; see e.g. Hogan et al. [45] and Saito et al. [78] for similar findings. Appendix B.1 considers other cost components and reaffirms the negative social gradient for total healthcare expenditures and prescription drugs. Meanwhile, like hospital expenditures, expenditures to primary care physicians plus other medical specialists display a negative social gradient at lower ages that switch to a positive gradient at higher ages.

Next, we consider the cumulative total healthcare expenditures used by a representative male and female, respectively, across a lifetime, i.e. using Eq. (4). The red lines on the left axis of Fig. 2 illustrate that the lowest socioeconomic group has the highest cumulative expenditures by each age of death. On the right axis of Fig. 2, we highlight the socioeconomic mortality gradient by calculating, from Eq. (5), the percentage of individuals within a socioeconomic group that die at each age. A larger percentage of both males and females in the lowest socioeconomic group (solid grey line) die prior to age 80. Conversely, the solid black line shows that a larger percentage of the highest socioeconomic group die between age 80 and 100+.

### Lifetime healthcare expenditures, and mortality

**Fig. 3:**
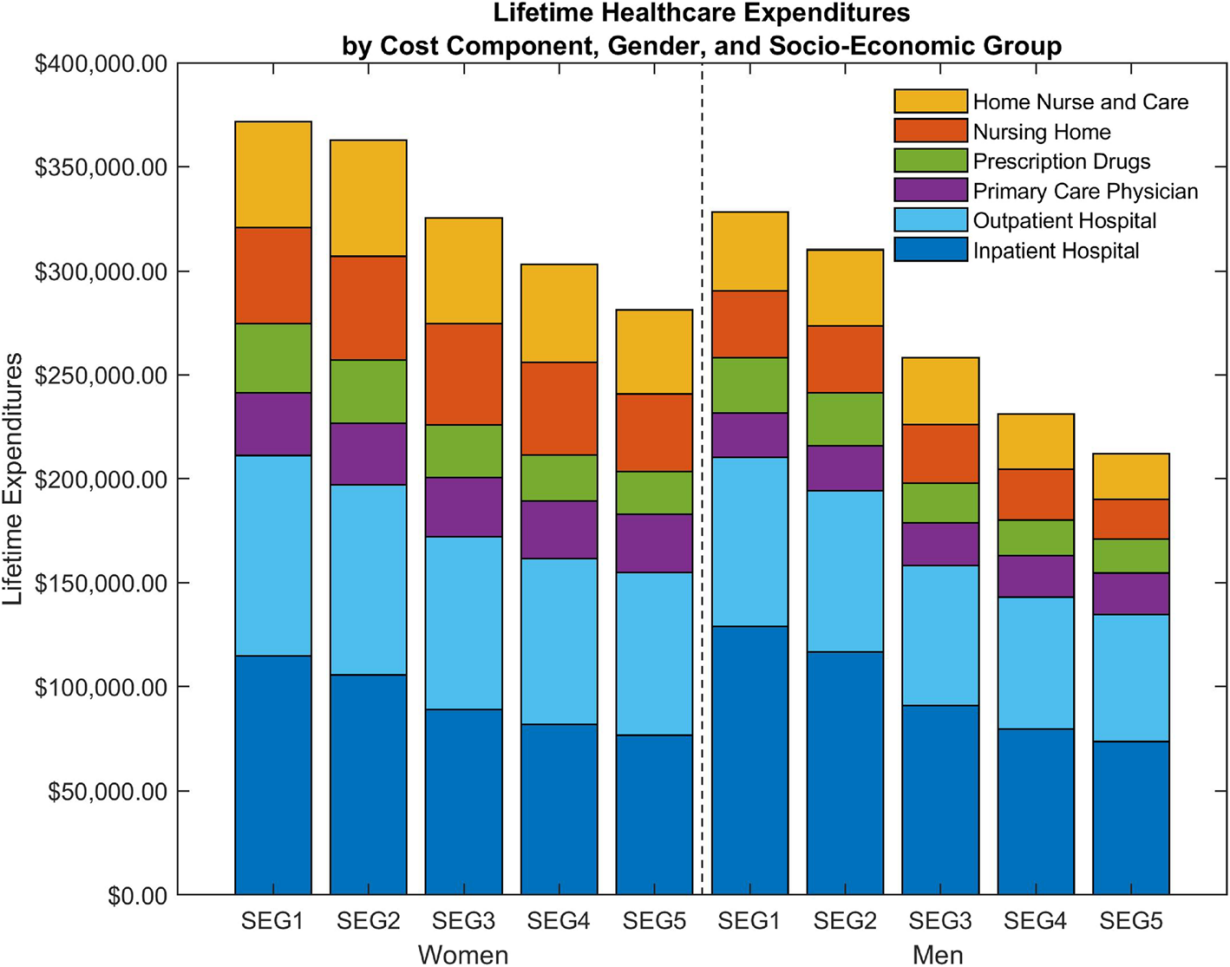
Lifetime healthcare expenditures: mortality by gender. Type of healthcare expenditure illustrated by different colors. Amounts are in 2012 USD

We add a lifetime perspective to the calculation by weighting the cumulative average healthcare expenditures from Fig. 2 by the percentage of deaths. This corresponds to the lifetime healthcare expenditures in Eq. (6). As a first step, we ignore socioeconomic mortality differences to illustrate the consequences of neglecting this dimension. Thus, we only use mortality rates that vary by gender as depicted by the dashed grey line in Fig. 2. A clear negative gradient arises in lifetime healthcare expenditures in Fig. 3, as previously confirmed for annual healthcare expenditures in the literature [16, 19]. Failing to account for the socioeconomic mortality differences gives rise to a negative social gradient in lifetime healthcare expenditures, i.e. a mortality-weighted extension of socioeconomic gradient in average annual healthcare expenditures in Fig. 1. As seen from the lifetime healthcare expenditure estimates in Table 1, corresponding to Fig. 3, males in the lowest socioeconomic group spend 54.6% [($328,067-$212,136)/$212,136] more on total healthcare in a lifetime perspective compared to the highest group. For females, the difference is 32.1% [($371,547-$281,199)/$281,199].

**Table 1:** Lifetime healthcare expenditures for Fig. 3

Lifetime healthcare expenditures: mortality by gender										
	Male					Female				
	SEG1	SEG2	SEG3	SEG4	SEG5	SEG1	SEG2	SEG3	SEG4	SEG5
Inpatient Hospital	129,029	116,648	91,092	79,662	73,708	114,870	105,807	89,028	81,804	77,006
	(1,122)	(1,033)	(841)	(792)	(727)	(859)	(878)	(694)	(703)	(833)
Outpatient Hospital	81,254	77,536	67,275	63,414	61,042	96,389	91,254	83,051	79,782	78,082
	(634)	(587)	(528)	(512)	(505)	(599)	(586)	(560)	(535)	(532)
Nursing Home	32,205	32,128	28,244	24,337	19,109	46,052	49,924	48,844	44,596	37,303
	(663)	(588)	(537)	(459)	(391)	(634)	(662)	(613)	(572)	(517)
Home Care + Home Nurse	37,489	36,569	32,099	26,566	21,984	50,901	55,651	50,762	47,008	40,392
	(732)	(653)	(604)	(536)	(487)	(724)	(770)	(698)	(683)	(604)
Prescription Drugs	26,634	25,560	19,255	17,143	16,315	33,047	30,395	25,298	22,175	20,422
	(133)	(136)	(119)	(111)	(100)	(134)	(131)	(122)	(105)	(100)
Primary Care Physician	21,455	21,684	20,424	20,077	19,978	30,288	29,763	28,476	27,834	27,995
	(79)	(78)	(75)	(73)	(73)	(85)	(86)	(87)	(80)	(86)
Total	328,067	310,125	258,390	231,200	212,136	371,547	362,793	325,459	303,199	281,199
	(2,035)	(1,888)	(1,667)	(1,546)	(1,479)	(1,882)	(1,955)	(1,749)	(1,645)	(1,705)

Lifetime healthcare expenditure estimates from Eq. (6). Note that mortality vary only by gender and age.

Amounts are in 2012 USD. Standard error in parenthesis

### Lifetime healthcare expenditures, socioeconomic groups, and mortality

**Fig. 4:**
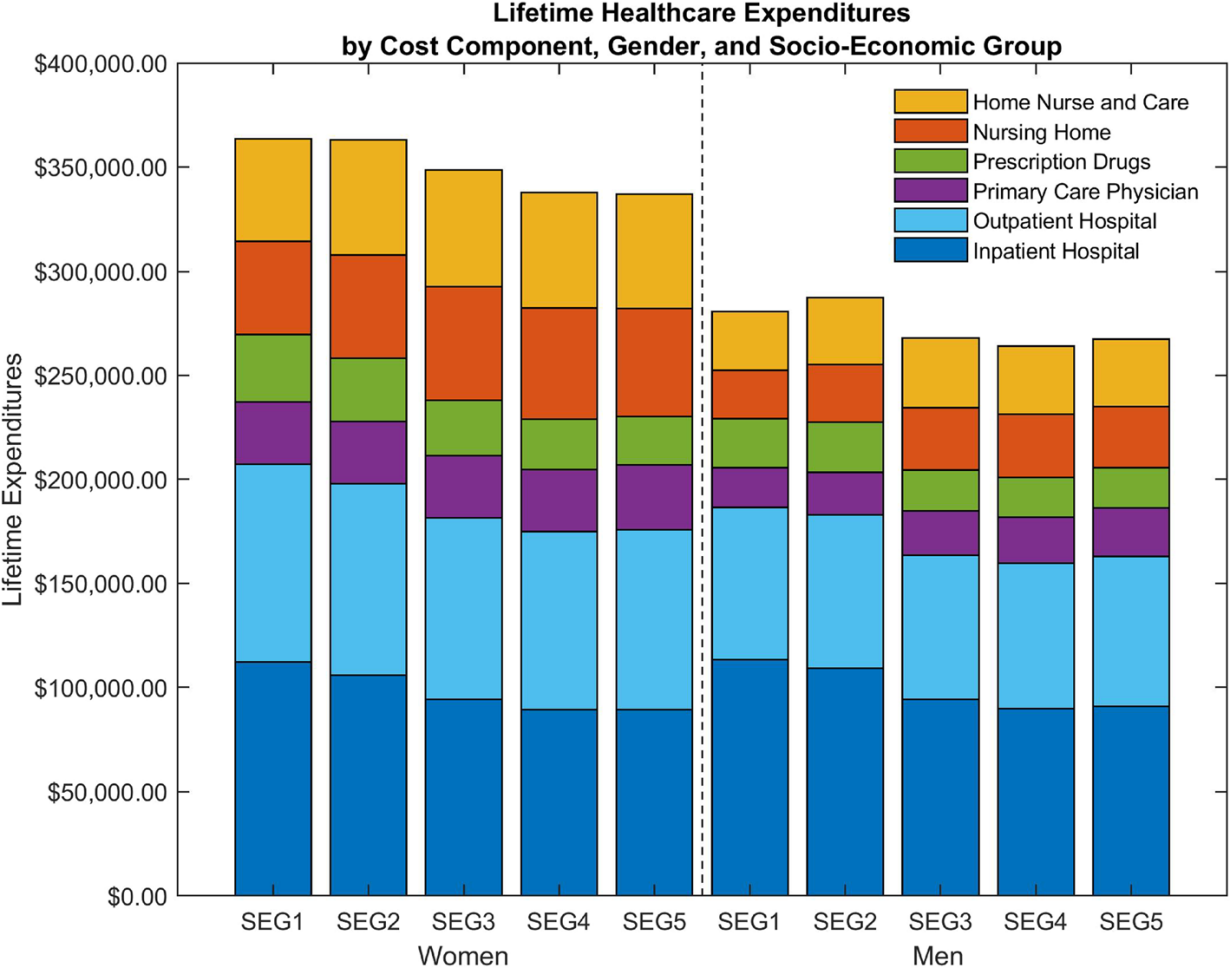
Lifetime healthcare expenditures: mortality by gender and socioeconomic group. Type of healthcare expenditure illustrated by different colors. Amounts are in 2012 USD

For our main results, we consider the healthcare expenditure and longevity gradients simultaneously. Estimating mean lifetime healthcare expenditures using the entire array of healthcare expenditures, socioeconomic mortality differences drastically diminish the social gradient in healthcare expenditures shown by the almost flat relationship between lifetime healthcare expenditure in Fig. 4. This relationship sharply contrasts the findings of a steep gradient in the average healthcare expenditures characterizing Fig. 1, the cumulative healthcare expenditures in Fig. 2, and the lifetime healthcare expenditures in Fig. 3. The lifetime healthcare expenditure estimates in Table 2, associated with Fig. 4, show how men in the lowest socioeconomic group on average spend $280,649 on healthcare across a lifetime, while men in the highest socioeconomic group spend $267,412. Meanwhile, females in the lowest and highest socioeconomic groups spend $363,637 and $337,077, respectively. Comparing the estimates in Tables 1 and 2, socioeconomic differences in lifetime expenditures decrease from 54.6% to 5.0% for males and from 32.1% to 7.9% for females. The gradient decreases, since mortality differences induce individuals in the lowest socioeconomic group to consume healthcare expenditures for fewer years than individuals in the highest socioeconomic group on average. However, a 5% or 8% difference may still be considered quantitatively meaningful.

Repeating the analysis of lifetime inpatient hospital expenditures by socioeconomic group, Table 2 confirms that both males and females in the lowest socioeconomic group have higher mean lifetime inpatient hospital expenditures than the highest socioeconomic group, i.e. $113,491 versus $90,960 for males and $112,441 versus $89,263 for females, respectively. The gradient increases from roughly 10% in Asaria et al. [5] to around 25% in our analysis.^5^ We uncover a novel additional negative gradient for lifetime prescription drugs expenditures. Males in the lowest socioeconomic group spend $23,531, i.e., 20.7% more than the highest socioeconomic group, which spends $19,495. For females, the difference is 38.3%. Finally, papers that compare gender-specific lifetime healthcare expenditure find a positive gradient for females [2, 10, 48, 96]. Table 2 confirms this finding and extends the result to hold for each socioeconomic group and cost component.

Contrary to existing knowledge, we uncover several novel positive gradients in lifetime healthcare expenditures in Table 2, specifically in nursing home, home care, and primary care physicians. For males, lifetime expenditures on primary care physicians are $19,119 for the lowest socioeconomic group, but 22% higher for the highest socioeconomic group, which spends $23,422. For females, the positive difference in nursing home costs is particularly pronounced, with lifetime expenditures of $44,656 for the lowest socioeconomic group, compared to $51,781 for the highest socioeconomic group. This finding corresponds to the positive gradient in Hurd et al. [48] for lifetime out-of-pocket nursing home use expenditures in the US. Yet, unlike the US, long-term care expenditures in Denmark are government-insured and are not subject to the ability to pay. The positive gradients partially discovered in this paper counteracts the aforementioned negative lifetime gradients towards more equal total lifetime healthcare expenditures. This finding is even more striking as the annual averages generally display negative gradients (see Appendix B.1).

**Table 2:** Lifetime healthcare expenditures for Fig. 4

Lifetime healthcare expenditures: mortality by gender and socioeconomic group										
	Males					Females				
	SEG1	SEG2	SEG3	SEG4	SEG5	SEG1	SEG2	SEG3	SEG4	SEG5
Inpatient Hospital	113,491	109,275	94,240	89,889	90,960	112,441	105,878	94,314	89,464	89,263
	(1,006)	(1,024)	(892)	(908)	(889)	(900)	(844)	(742)	(819)	(905)
Outpatient Hospital	73,066	73,607	69,459	69,896	71,931	95,015	92,051	87,206	85,488	86,480
	(644)	(602)	(608)	(606)	(622)	(639)	(579)	(602)	(599)	(618)
Nursing Home	23,241	27,625	29,682	30,517	29,185	44,656	49,538	54,306	53,363	51,781
	(568)	(648)	(712)	(758)	(717)	(842)	(890)	(925)	(918)	(883)
Home Care + Home Nurse	28,202	32,322	33,497	32,596	32,417	49,307	55,177	56,011	55,506	54,942
	(647)	(697)	(764)	(802)	(810)	(911)	(1,019)	(964)	(972)	(996)
Prescription Drugs	23,531	24,123	19,915	19,089	19,495	32,363	30,484	26,751	24,181	23,403
	(163)	(160)	(147)	(145)	(147)	(179)	(169)	(159)	(149)	(137)
Primary Care Physician	19,119	20,525	21,084	22,070	23,422	29,855	30,007	29,944	29,903	31,209
	(107)	(112)	(110)	(116)	(126)	(127)	(118)	(116)	(114)	(118)
Total	280,649	287,477	267,878	264,057	267,412	363,637	363,134	348,532	337,905	337,077
	(2,185)	(2,279)	(2,302)	(2,349)	(2,345)	(2,533)	(2,550)	(2,537)	(2,500)	(2,525)

Lifetime healthcare expenditure estimates from Eq. (6). Note that mortality vary by both gender and socioeconomic group. Amounts are in 2012 USD. Standard error in parenthesis

### Statistical tests

Our main results illustrate how accounting for mortality differences sharply decrease the socioeconomic differences in lifetime healthcare expenditures, i.e. numerically. Yet, to rigorously test whether lifetime healthcare expenditures in Fig. 4 differ across socioeconomic groups, Table 3 applies the Welch [89] test of equal means from Eq. (7) for the highest and lowest socioeconomic groups. That is, a null hypothesis of equal lifetime healthcare expenditures by gender and expenditure component $H_0: LHE_{SEG1,g,t} = LHE_{SEG5,g,t}$. This approach is new in the literature. With a sample size of more than 300,000 individuals in each socioeconomic group, we strongly reject the null of equal mean total lifetime healthcare expenditures across socioeconomic groups with a *p*-values of 0.000 for both males females.^6^ Thus, even though the socioeconomic differences in lifetime healthcare expenditures are greatly reduced, the negative social gradient in total lifetime healthcare expenditures prevails and remains statistically significant.

Using the Welch [89] test for lifetime inpatient hospital expenditures, Table 3 strongly rejects the equality of the means for both males and females with *p*-values of 0.000 and hereby further strengthens the result in Asaria et al. [5] with statistical rigor. In addition, the positive lifetime gradients in expenditures to nursing homes, home care, and primary care physicians are statistically significant, and we reject that the positive gradient arises from sampling error. Performing the test for other socioeconomic groups, we find the same results. Only for a few cases are we unable to reject the null, typically for adjacent socioeconomic groups (see Appendix B.2).

**Table 3:** *P*-values from Welch test of equal lifetime healthcare expenditures

*P*-values from Welch test		
	Males	Females
Total	0.000	0.000
Inpatient Hospital	0.000	0.000
Outpatient Hospital	0.000	0.000
Nursing Home	0.000	0.000
Home Care + Home Nurse	0.000	0.000
Prescription Drugs	0.000	0.000
Primary Care Physician	0.000	0.000

Test of equal lifetime healthcare expenditures between the lowest and highest socioeconomic groups, SEG1 and SEG5, respectively, using the Welch [89] test in Eq. (7).

$$H_0: LHE_{SEG1,g,t} = LHE_{SEG5,g,t}$$

### Robustness estimates

To contest our results, we perform several robustness analyses. First, to resemble the methodology in the paper most similar to ours [5], we lower the top-coded age from 100 to 85 and estimate lifetime healthcare expenditures in Appendix C.1. As seen from Table 4, the level of total healthcare expenditures decreases as fewer years are available to consume healthcare expenditures. The gap in lifetime healthcare expenditures between the highest and lowest socioeconomic groups increases by 18.4 percentage points for males and 16.3 percentage points for females, which illustrates the importance of accounting for socioeconomic differences in healthcare expenditures and mortality from age 85 to 100. Hence, setting a low top-coded age inflates the socioeconomic gradient in total lifetime healthcare expenditures.

Second, our main estimates of lifetime healthcare expenditures use the average of healthcare expenditures and mortality by age. In contrast, Asaria et al. [5] calculate averages in 5-year age bands to smooth the age distributions of healthcare expenditures and mortality. To assess the robustness of our results towards this difference, we estimate lifetime healthcare expenditures using; 3-year age bands, 5-year age bands, and a smoothing-spline, in Appendix C.2. Our conclusion remains unchanged, and mean lifetime healthcare expenditures differ by less than $1,500, and the test results remain unchanged. See Table 4 for the 5-year age band result.

Third, to assess the impact of our choice of socioeconomic measure on our findings, we use educational attainment as an alternative measure of socioeconomic status [48, 59]. We use ISCED codes [86] to classify individuals into the following groups: basic, short, medium, and long education. The four education groups consume almost identical lifetime healthcare expenditures, and we are unable to reject the null of equal lifetime healthcare expenditures for females but reject it for males; see Table 4 and Appendix C.3 for additional results. Generally, using education as a socioeconomic measure raises concerns about cohort effects, i.e. the fraction of the population with a given level of education changes over time. In particular, the sparse observations of highly educated individuals at extreme ages result in a high bootstrapped standard error. Despite these fluctuations, the highest affluence-based group has the highest percent of higher educations and the least of basic education by cohorts and vice versa for the lowest affluence group (see Appendix A.3).

**Table 4:** Robustness estimates of total lifetime healthcare expenditure

	Males			Females		
	SEG1	SEG5	*P*-value	SEG1	SEG5	*P*-value
Main	280,649	267,410	0.000	363,637	337,077	0.000
	(2,185)	(2,345)		(2,533)	(2,525)	
Top-code age 85	253,892	205,193	0.000	296,788	240,048	0.000
	(1,647)	(1,330)		(1,635)	(1,353)	
5-year age band	281,183	268,131	0.00005	364,362	337,342	0.000
	(2,188)	(2,346)		(2,533)	(2,522)	
Education Groups	264,833	248,945	0.003	321,399	312,318	0.394
	(1,466)	(5,137)		(1,384)	(10,572)	

Robustness estimates of total lifetime healthcare expenditures and associated Welch [89] tests of equal lifetime healthcare expenditures between the lowest and highest socioeconomic groups, SEG1 and SEG5, respectively. For Education groups, SEG1 refers to individuals with the least education and SEG5 to individuals with the longest education. $H_0: LHE_{SEG1,g,t} = LHE_{SEG5,g,t}$

Bootstrapped standard errors in parenthesis

## Discussion

Despite the challenges in comparing healthcare systems, the size of our estimates in Table 2 is conformable with existing estimates of lifetime healthcare expenditures. Alemayehu and Warner [2] use similar cost components as this paper for a large healthcare insurer in the US but disregard the socioeconomic dimension. They find that throughout a lifetime, females spend about $361,000, whereas males spend $268,000. Using Dutch data, Wong et al. [96] find that females spend $211,000 over a lifetime, whereas males spend $177,000.^7^ As this study includes neither long-term care nor a socioeconomic dimension, we exclude these components from our estimates to enable a comparison. We now find that females spend $243,140 on healthcare during a lifetime. The corresponding number for males is $213,638.

Like the majority of the existing literature [2, 5], our estimates of lifetime healthcare expenditures assume static patterns of healthcare consumption, longevity, and morbidity. Below, we discuss the effect of these three assumptions on lifetime healthcare expenditures. Average healthcare expenditures are not constant but may change over time [44, 61] due to, for example, medical innovations [13]. Dickman et al. [28] showed that Americans in all income quintiles had steady, but different, growth rates in per capita medical expenditures from 1963 to 2011. Since 2004, the age- and health-adjusted expenditure differences between the highest and lowest income quintile increased from zero to $1,743. Meanwhile, evidence from the EU finds very limited growth in age-specific average healthcare expenditures [94]. Increases in healthcare expenditures suggest that individuals who survive from age 30 and onward consume a socioeconomic-specific higher amount of average healthcare expenditures in each subsequent year alive. If this is the case, our analysis likely underestimates future lifetime healthcare expenditures. For future work, we propose to capture these changes and their effect on lifetime healthcare expenditures by using a predictive model that allows socioeconomic specific changes in the age distribution of average healthcare expenditures. Alternatively, historical socioeconomic variations in lifetime healthcare expenditures could be explored using long panels capturing cohort effects as suggested in the life cycle consumption literature ([27], Chapter 6). Unfortunately, our full panel of healthcare expenditure data stretches to three years at most, which is unsatisfactory for both forecasting healthcare expenditures and historical analyses.

When forecasting healthcare expenditures, changing morbidity patterns can influence healthcare expenditure trends. In particular, populations tend to age with better health and live fewer years with morbidities than previous generations - the so-called compression of morbidity [40, 41]. Evidence suggests that the intensity of healthcare usage decreases among the young, but increases among the oldest [95], and the less affluent tend to age more quickly [18, 83]. At death, a higher number of morbidities are observed, particularly within the lowest socioeconomic groups [76], and over time, healthcare expenditures have increased in the last years of life [43]. These changes suggest that the age distribution of average healthcare expenditures in Fig. 1 shifts right over time, which potentially could affect lifetime healthcare expenditure estimates in both directions. However, estimation of these effects remains for future research with longer time series.

Changes in morbidity patterns affect not only healthcare expenditures but also life span equality [1], longevity, and mortality rates [59, 65] which have improved historically. For instance, disease-specific technological innovations have had different mortality effects across the socioeconomic distribution [21]. Failing to capture mortality improvements implies that individuals consume healthcare expenditures for too few years, which could underestimate lifetime healthcare expenditures. Meanwhile, considering mortality trajectories in isolation might be too simplistic as any simultaneous trends in average healthcare expenditures and longevity, due to, say, underlying morbidity patterns, are omitted. To capture the joint effects of morbidity patterns, healthcare expenditure changes, and longevity trends on lifetime healthcare expenditures, we suggest that future research should forecast both average healthcare expenditures and mortality with a simultaneous model that includes socioeconomic differences. We do, however, note that this added complexity requires future healthcare expenditures to be discounted by intertemporal preferences suggesting that lifetime healthcare expenditure estimates might remain unchanged.

A related point on individual morbidity is the role of individuals’ health around retirement, specifically, as we fix our main socioeconomic measure at age 65. The healthiest individuals within lower socioeconomic groups potentially outlive their peers, thereby skewing the health profile within these groups and making them appear relatively healthier, which may affect our findings. However, recent evidence from Danish register data reveals that individuals in the lowest socioeconomic groups consistently experience poorer health outcomes compared to those in higher socioeconomic groups at older ages, as they are more likely to transition to poor health states and less likely to recover compared to their higher-socioeconomic status counterparts [53]. Consequently, even in old age, lower socioeconomic groups exhibit significantly worse health outcomes compared to those in higher socioeconomic groups.

## Conclusion

This paper reexamines the negative social gradient in healthcare expenditures, but, unlike existing literature, accounts for socioeconomic mortality differences and uses full-population data on complete healthcare expenditures. We estimate lifetime healthcare expenditures across five socioeconomic groups using uniquely detailed population-wide Danish register data. From age 30 to death, females in the lowest and highest socioeconomic groups spend, on average, $363,637 and $337,077 on healthcare, respectively, while males spend $280,649 and $267,412. Importantly, despite lower socioeconomic groups contributing a disproportionately large share to total healthcare expenditures each year, our findings emphasize an essential equality in the utilization of healthcare resources over time. The longer lifespan during which individuals from higher socioeconomic groups use healthcare services narrows the gap in lifetime healthcare expenditures. Therefore, from a fairness perspective regarding healthcare expenditures, individuals from all socioeconomic groups utilize healthcare resources equitably throughout their lifetimes.

As a key contribution, we demonstrate that, once socioeconomic mortality differences are taken into account, the negative social gradient in lifetime healthcare expenditures decreases by 24.2 percentage points for females and 49.6 percentage points for males. Although this is an immense drop in the socioeconomic inequality, some socioeconomic differences in lifetime healthcare expenditures persist. Applying a Welch [89] test, we find that males in the lowest socioeconomic group have a significantly larger mean total lifetime healthcare expenditure of 5.0%. The equivalent number for females is a 7.9% difference, which is also statistically significant. We document a novel positive gradient in lifetime nursing home expenditures and home care expenditures, with the highest socioeconomic group spending the most. The negative gradients of the major cost components, hospital care and long-term care, equalize total lifetime healthcare expenditures, emphasizing the importance of considering a complete array of cost components.

Disappointingly, the social gradients in health and longevity persist even in a universal healthcare system, such as the Danish, even though all socioeconomic groups have equal access. Policymakers aiming for equality in health should be mindful of the simultaneous effects that health policies can have on healthcare expenditures and longevity. When the health level of a lower socioeconomic group improves, average healthcare expenditures and mortality rates decline to resemble those of more affluent groups. Since all socioeconomic groups have the same level of lifetime healthcare expenditures, lifetime expenditures could remain unchanged. Meanwhile, the lifetime utility of the socioeconomic group would likely improve as better health increases life expectancy [56] and correlates positively with wellbeing [81, 82].

From a policy point of view, our results have important implications. If healthcare expenditures are postponed to older ages due to health improvements, the timing of the financial burden of healthcare changes. Future national healthcare expenditures increase, and current expenditures decline, offering policy-relevant suggestions for how the costs of health interventions could be financed.

## Supplementary information

Supplementary Material 1.

## Data Availability

Individual-level information from the administrative registers is confidential to the Danish Administrative Procedures (Section 27) and the Danish Criminal Code (Section 152), and data cannot be made publicly available. The data used for this analysis are available upon submission of an application to Statistics Denmark, https://www.dst.dk/en. Restrictions may apply to the availability of data. Upon request, the authors will assist in replicating study results.

## Footnotes

1 OECD data categories HC3.1 and HC3.2 from are used.

2 A feature of the Cairns et al. [8] affluence measure is that individuals may change socioeconomic groups over time which ensures similar cohort sizes across age, unlike for instance education. As seen in Appendix A.2, around 70% of the population remained within the same socioeconomic group between 2011 and 2012 and approximately 25% switch to an adjacent group. Hence, the measure is relatively stable. Meanwhile, most of our analyses use the 2012 socioeconomic groups and individuals do not switch groups.

3 Further, using the Welch test allows tests of our lifetime healthcare expenditure measure encompassing both socioeconomic differences in healthcare expenditures and mortality as opposed to tests such as Chiang [15] designed for only life expectancy.

4 The average cost of a birth ranges between approximately USD 6,800 for a cesarean or firstborn and USD 3,800 for subsequent births [73].

5 As seen in Appendix C.1, lowering the top-coded age to age 85, the top-coded age in Asaria et al. [5], increases the level of lifetime healthcare expenditures, but simultaneously more than doubles the percentage point difference in lifetime healthcare expenditures. Hence, accounting for the socioeconomic differences in mortality and inpatient hospital expenditures between ages 85 and 100+ shrinks the socioeconomic gap in lifetime inpatient hospital expenditures.

6 We rely on a 5% significance level to draw our conclusions. Multiple testing correction have been suggested to arrive at the desired overall significance level across all tests. The most conservative correction, the Bonferroni [7] correction, reduces the significance level of each test to $5\%/140=0.00036$, where 140 is the number of tests performed for our main analysis. Our main conclusions in “Results” section remain unchanged, even when the conservative Bonferroni [7] correction is applied. We acknowledge other approaches, e.g., Romano and Wolf [74, 75], which reduced power to a lesser degree; however, at infeasibly high computational costs in our setting.

7 We use the 2012 1.28$/€ exchange rate from FRED, Federal Reserve Bank of St. Louis [36] and a Dutch projection rate between 2005 to 2012 of size 1.39 from OECD [70].
