# Supplementary material for: Lifetime healthcare expenditures across socioeconomic groups
Source: BMC Public Health. 2024 Oct 9;24:2751. doi: 10.1186/s12889-024-20209-1 (PMC11463145; doi:10.1186/s12889-024-20209-1)
Supplement: Supplementary file 1 — Supplementary Material 1. [file 12889_2024_20209_MOESM1_ESM.pdf]

Online Appendix for Lifetime Healthcare Expenditures Across Socioeconomic Groups

Malene Kallestrup-Lamb*

Aarhus University and PeRCent

Alexander O. K. Marin†

Aarhus University

September 25, 2024

*Fuglesangs Allé 4, 8210 Aarhus V, Denmark. Email: mkallestrup@econ.au.dk

†Corresponding Author. Campusvej 55, 5230, Odense M, Denmark. Phone: +45 6550 9606. Email: aokm@sam.sdu.dk. Current affiliation: University of Southern Denmark

A Data

A.1 Summary Statistics

Table A.1 shows the mean and standard deviation of selected variables using samples of everyone, males, females, and each of the five socioeconomic groups. Total expenditure is the sum of all expenditures to hospitals, primary care physicians, prescription drugs, and long-term care. Hospital expenditures include expenditures on in- and outpatient care, and long-term care accumulates expenditures on nursing homes, home care, and home nurses.

Females spend more on healthcare than males overall. Long-term care is particularly higher for females due to, for example, the longer average lifespan of females. The longer lifespan is also reflected in females' higher average age. Comparing healthcare expenditures across socioeconomic groups reveals that the highest socioeconomic group, SEG 5, spends less on healthcare than the lowest socioeconomic group, SEG 1. This emphasizes the negative social gradient in healthcare expenditures. For long-term care, the intermediate socioeconomic groups, SEG 2 and SEG 3, spend the most on long-term care, and the highest group spends the least. Note that the standard deviation of healthcare expenditures is larger for the lowest socioeconomic groups, indicating that expenditures are more dispersed for these groups.

Average income and wealth are higher for males than females, and income and wealth increase with the socioeconomic group, that follows from the definition of the groups. The average age increases with the socioeconomic group since we lock in socioeconomic groups at age 67, and at age 67, higher socioeconomic groups, on average, have a longer life expectancy.

The group size of the high socioeconomic groups is slightly larger than for low socioeconomic groups. Though evenly sized before age 67, the high socioeconomic groups have a longer average lifespan. When we fix socioeconomic groups when individuals turn 67, as described in Cairns et al. (Cairns et al., 2019), the mortality differences cause the relative size of cohorts in the highest socioeconomic group to increase. Consequently, the size of the highest socioeconomic group has a slightly larger sample size, N.

Table A.1: Summary Statistic

Summary Statistics

				Males					Females				
	All	Female	Male	SEG 1	SEG 2	SEG 3	SEG 4	SEG 5	SEG 1	SEG 2	SEG 3	SEG 4	SEG 5
Total Expenditures	5,385	5,912	4,831	5,878	5,682	4,667	4,220	3,989	6,739	6,402	5,775	5,544	5,340
	(15,909)	(16,181)	(15,599)	(17,826)	(17,401)	(15,264)	(14,116)	(13,271)	(17,107)	(16,890)	(15,340)	(15,578)	(16,179)
Total Hospital Expenditure	3,315	3,402	3,223	4,114	3,801	3,034	2,742	2,612	4,104	3,761	3,251	3,060	2,970
	(12,451)	(12,017)	(12,890)	(15,488)	(14,563)	(12,159)	(11,300)	(10,603)	(13,396)	(12,813)	(10,749)	(10,958)	(12,150)
Inpatient Hospital Expenditures	1,781	1,727	1,838	2,471	2,236	1,704	1,488	1,401	2,177	1,952	1,625	1,505	1,448
	(9,487)	(8,927)	(10,040)	(12,482)	(11,648)	(9,274)	(8,508)	(7,762)	(9,966)	(9,598)	(7,481)	(7,960)	(9,516)
Outpatient Hospital Expenditures	1,533	1,675	1,385	1,642	1,565	1,330	1,254	1,211	1,927	1,809	1626	1555	1521
	(6,221)	(6,408)	(6,015)	(6,767)	(6,403)	(5,853)	(5,638)	(5,500)	(7,028)	(6,628)	(6,201)	(6,127)	(6,127)
Doctor Expenditures	486	561	407	423	431	404	398	398	598	582	555	542	546
	(662)	(701)	(608)	(623)	(658)	(604)	(581)	(586)	(740)	(730)	(691)	(668)	(682)
Medical Expenditure	450	493	405	519	505	372	332	321	639	573	471	416	389
	(1,221)	(1,193)	(1,248)	(1,367)	(1,452)	(1,242)	(1,118)	(1,045)	(1,408)	(1,302)	(1,170)	(1,034)	(1,011)
Long-term Care	1,135	1,457	797	823	945	857	748	659	1,399	1,486	1,497	1,526	1,435
	(8,263)	(9,314)	(6,975)	(6,854)	(7,400)	(7,470)	(6,864)	(6,437)	(9,009)	(9,271)	(9,334)	(9,751)	(9,371)
Nursing Home Expenditures	526	690	355	352	413	383	347	302	643	683	722	737	690
	(4,465)	(5,099)	(3,675)	(3,661)	(3,958)	(3,816)	(3,631)	(3,390)	(4,926)	(5,074)	(5,215)	(5,269)	(5,100)
Home Care Expenditures	559	708	403	426	480	435	370	333	689	734	714	733	697
	(5,439)	(6,013)	(4,758)	(4,616)	(5,013)	(5,236)	(4,622)	(4,395)	(5,899)	(5,970)	(5,906)	(6,364)	(6,033)
Home Nurse Expenditure	49	60	38	45	53	39	32	24	66	69	61	56	48
	(460)	(508)	(403)	(427)	(477)	(415)	(377)	(313)	(527)	(547)	(519)	(498)	(451)
Income	341,339	297,096	387,853	189,300	266,219	326,559	407,548	737,791	171,618	236,719	277,734	327,296	468,581
	(632,527)	(323,694)	(840,442)	(116,060)	(105,198)	(120,984)	(174,807)	(1,788,627)	(83,266)	(73974)	(94,648)	(114,386)	(655,300)
Wealth	745,780	657,620	838,466	227,375	364,450	544,168	806,896	2,208,452	246,613	311,838	446,355	631,003	1,622,750
	(7,403,731)	(4,774,632)	(9,405,343)	(531,692)	(529,193)	(687,233)	(999,980)	(20,718,058)	(611,259)	(448,872)	(559,836)	(786,615)	(10,433,181)
Male	0,49	0	1	1	1	1	1	1	0	0	0	0	0
	(0,5)	(0)	(0)	(0)	(0)	(0)	(0)	(0)	(0)	(0)	(0)	(0)	(0)
Age	55	56	54	54	54	55	55	55	56	56	56	56	57
	(15)	(16)	(15)	(14)	(14)	(15)	(15)	(15)	(15)	(15)	(16)	(16)	(16)
N	3,592,458	1,840,271	1,752,187	338,192	341,880	345,972	350,031	355,205	360,826	359,283	362,519	367,673	373,603

Amounts are in 2012 USD. Table shows mean values. Standard deviations are in parentheses.

A.2 Changing Socioeconomic Groups

Figure A.1 shows the percentage of individuals that change socioeconomic group between 2011 and 2012 by age. Negative change refers to a move from a higher to a lower socioeconomic group and positive changes refer to the reverse. Most individual stay within the same socioeconomic group or change to an adjacent group. Larger changes are more infrequent but do happen. Changes happen more often at lower ages suggesting more volatile incomes and lower levels of wealth.

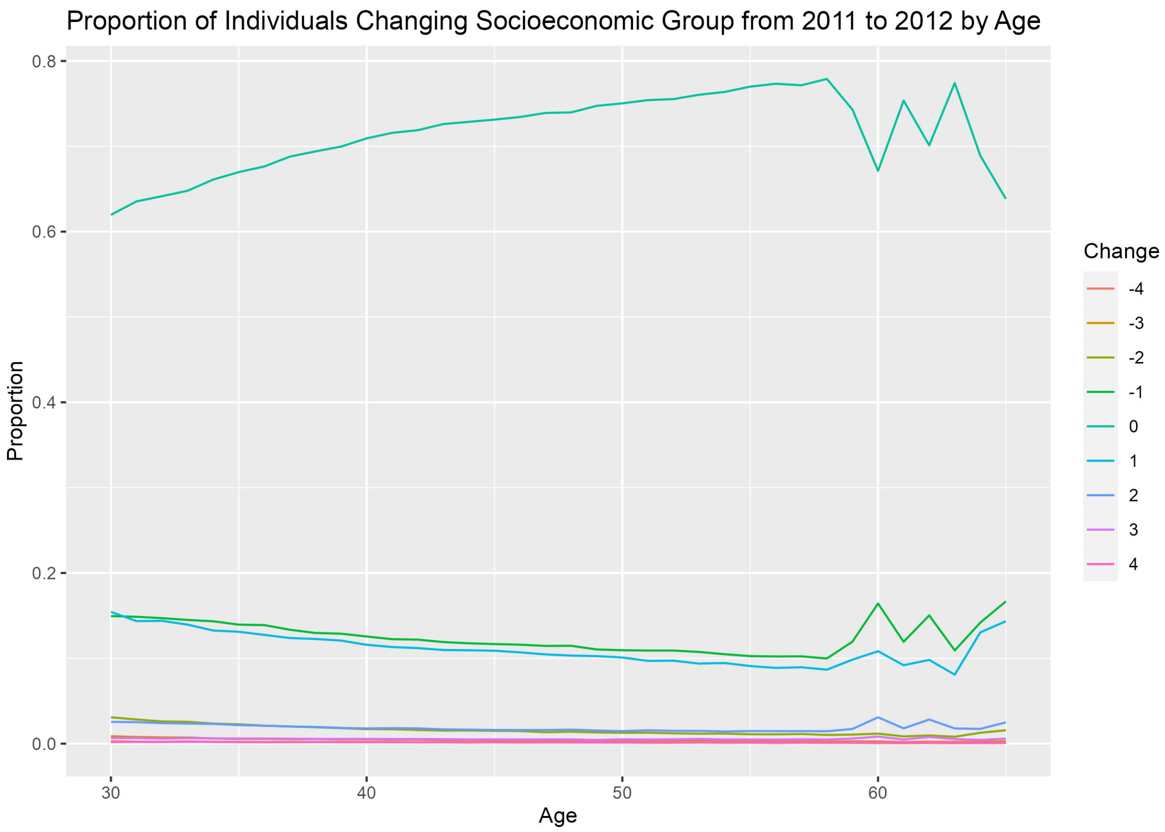

Figure A.1: The percentage of individuals that change socioeconomic groups between 2011 and 2012.

A.3 Education Group Definition

In robustness checks, we use educational attainment to determine socioeconomic groups instead of the affluence-based socioeconomic groups (Cairns et al., 2019) used in the paper. We divide our sample into four socioeconomic groups based on educational information from the Danish education registers described in (Jensen and Rasmussen, 2011). Using the international ISCED education codes (UNESCO, 2012), the first group, *basic*, has ISCED level 3 or less, which corresponds to high school or less than years of education as the highest educational attainment. The second group

includes individuals with practical vocational training, *voc*, or theoretical short higher education, *she*, which requires between 14 and 15 years of education at ISCED 4 or ISCED 5 level. The third group has medium higher education, *mhe* with 15 to 17 years of education at the ISCED 6 level. Lastly, with 17 or more years of education at ISCED 7 and ISCED 8, we define the long higher education, *lhe*, group.

We now discuss some of the advantages of using our affluence-based socioeconomic status measure compared to education-based socioeconomic groups. First, the affluence-based socioeconomic groups have a consistent sample size in the age dimension, which increases the homogeneity of the socioeconomic groups for each age, as opposed to education groups, where group sizes fluctuate wildly in the age dimension, as shown in Figure A.2. Using education as a socioeconomic measure hence inadvertently compares groups that are time-inconsistent, as discussed by (Bound et al., 2015). Second, our data only has educational information for individuals below age 90, whereas the affluence measure runs to age 100+. This difference in maximum age proves important as a gradient in lifetime healthcare expenditures increases when we set the highest age to 85 using our affluence-based socioeconomic groups to resemble Asaria et al. (2016). This can be seen by comparing Figure 4 in the paper and lifetime estimates top-coded at age 85 in Figure A.11. Lastly, for the older population, the educational measure lacks quality and has many missing values, which excludes important observations.

A.3.1 Cohort Size for Education Groups

Figure A.2 shows the fraction of a cohort in 2012 that belongs to one of the four education groups defined in section A.3. The graph illustrates the challenges with education group homogeneity in the age dimension, namely, that the basic education group is larger and more diverse for cohorts age 90 compared to the basic education group at age 30. Similarly, more individuals at age 30 have a long higher education, and since the group contains more individuals, the group is more heterogeneous than the group of individuals with a long higher education at age 90. At age 90, the group only includes the few individuals at the far tail of the socioeconomic distribution. Hence, the distribution of socioeconomic status shifts across time/cohorts when education measures socioeconomic status. Consequently, we end up comparing parts of the population that are inconsistent across cohorts.

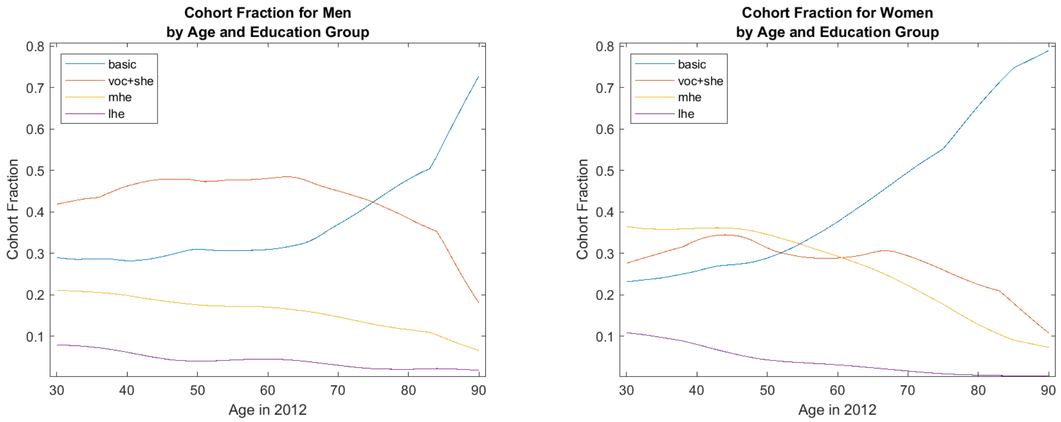

Figure A.2: Smoothed fraction of entire cohort in each of the four education groups in 2012 for males (left) and females (right).

A.3.2 Relationship Between Education and Affluence-based Socioeconomic Groups

Figure A.3 show the percentage of individual in lowest (solid) and highest (dotted) socioeconomic group that belong to the different educational groups by age for females (left) and males (right). A higher percentage og individuals in the lowest socioeconomic group, SEG1, have a basic level of education while long higher education (lhe) and medium higher education (mhe) is more frequent in the highest socioeconomic group, SEG5, i.e., higher educational status correlate positively with higher affluence-based socioeconomic status. In terms of vocational training and short higher education (voc+she) the socioeconomic group with the highest percentage change across ages which reflect cohort effect, among other things.

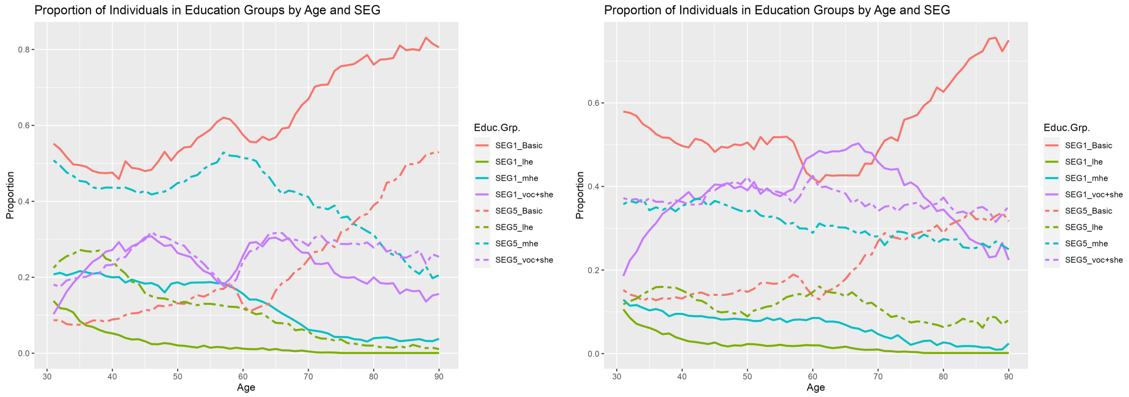

Figure A.3: Percentage of individuals within each educational group by age and socioeconomic group for females (left) and males (right).

B Additional Results

B.1 Analysis of Average Healthcare Expenditures

In this section, we analyze average healthcare expenditures by age, socioeconomic group, and cost component. Figure A.4 shows the age distribution of total healthcare expenditure for males and females aged 30 through 100+ across socioeconomic groups in 2012. The lowest socioeconomic group, SEG 1, spends the most on healthcare at most ages, while the highest socioeconomic group, SEG 5, generally has the lowest expenditures. This holds for both males and females. Average healthcare expenditures increase with age for all socioeconomic groups. These patterns reflect the fact that expenditures increase with age , more die at higher ages, and expenditures increase up until death, see, e.g., (Seshamani and Gray, 2004).

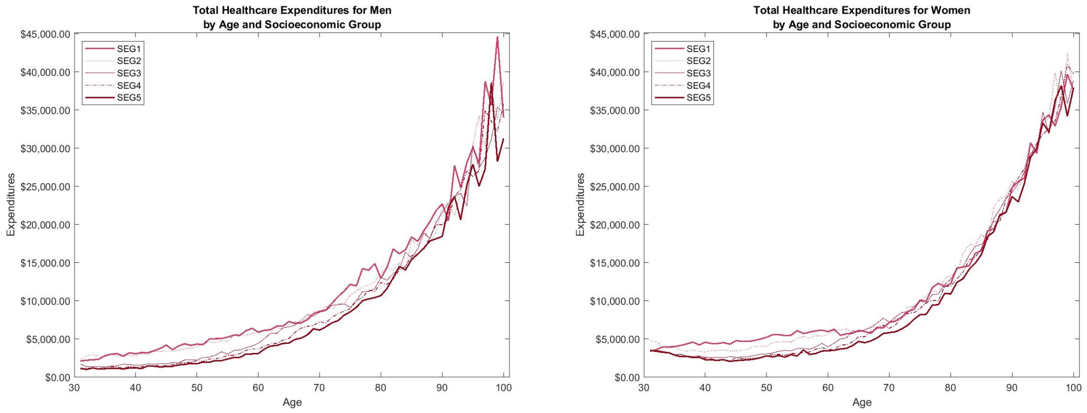

Figure A.4: Total expenditures on healthcare for males (left) and females (right) across socioeconomic groups in 2012.

Focusing on inpatient hospital expenditures in Figure A.5 reveals that the lowest socioeconomic group, SEG1, on average spends most on inpatient care up to around age 85 for males and age 80 for females. Hereafter, the difference in expenditures disappears, and data becomes noisier.

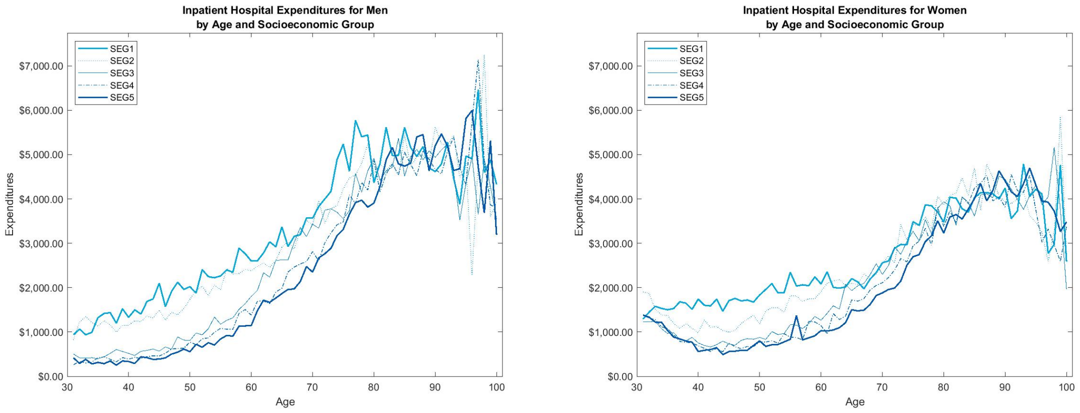

Figure A.5: Inpatient hospital expenditures for males (left) and females (right) across socioeconomic groups in 2012.

In Figure A.6, the expenditure pattern resembles that for inpatient care in Figure A.5. Up to around age 80, the lowest socioeconomic group, SEG1, spends the most for both males and females. Interestingly, however, from around age 80 to 100, the highest socioeconomic group, SEG5, spends the most on average.

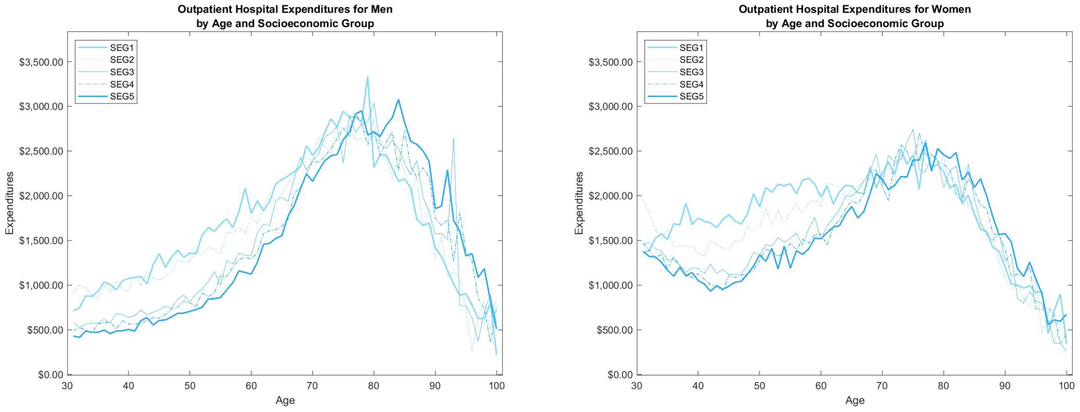

Figure A.6: Outpatient hospital expenditures for males (left) and females (right) across socioeconomic groups in 2012.

As seen in Figure A.7, males in the lowest socioeconomic group, SEG1, spend the most on primary care physicians from ages 30 to 70. However, at ages 70 to 100, this pattern flips, and the highest socioeconomic group, SEG5, spends the most. For females, a similar shift happens, but at age 65.

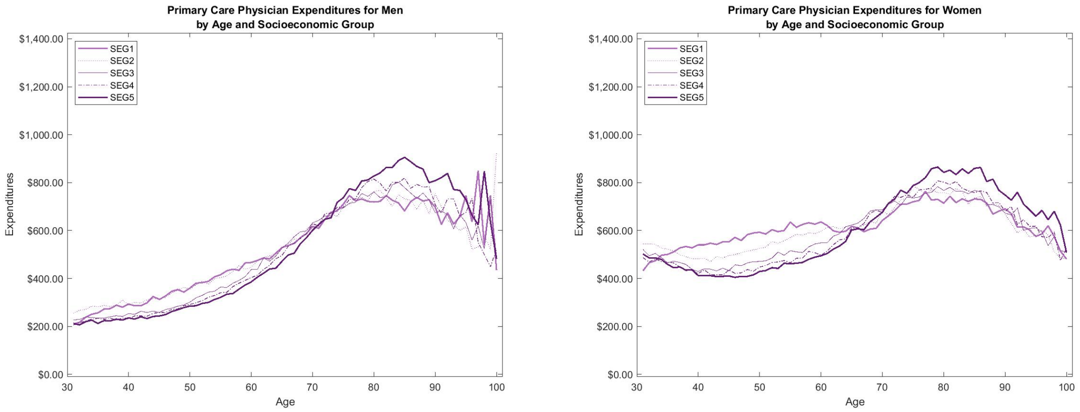

Figure A.7: Total physician expenditures for males (left) and females (right) across socioeconomic groups in 2012.

Expenditures to prescription drugs in Figure A.8 reveal that for all ages, males and females in the lowest socioeconomic group, SEG1, have the highest average expenditures, whereas males in the highest socioeconomic group, SEG5, spend the least.

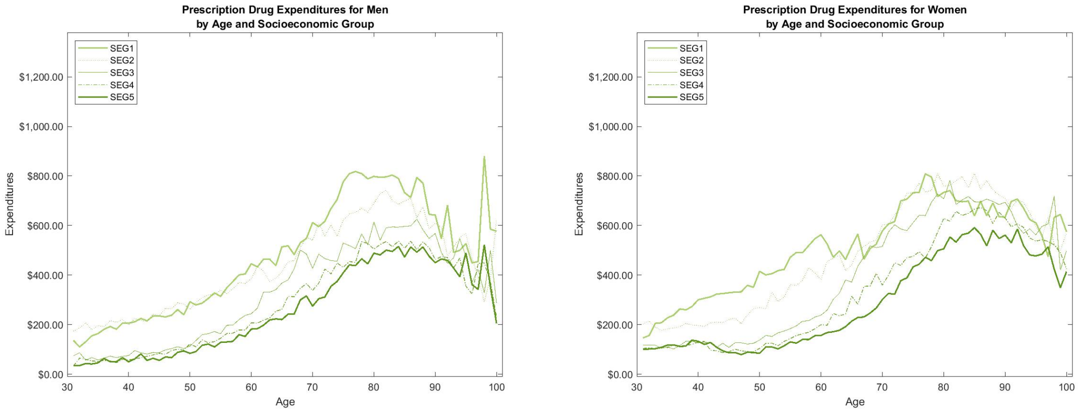

Figure A.8: Total prescription drug expenditures for males (left) and females (right) across socioeconomic groups in 2012.

Expenditures to nursing homes in Figure A.9 and expenditures to home care and home nurses in Figure A.10 are highest for the lowest socioeconomic group, SEG1, for females after age 65. However, for males, the difference in expenditures between socioeconomic groups is small.

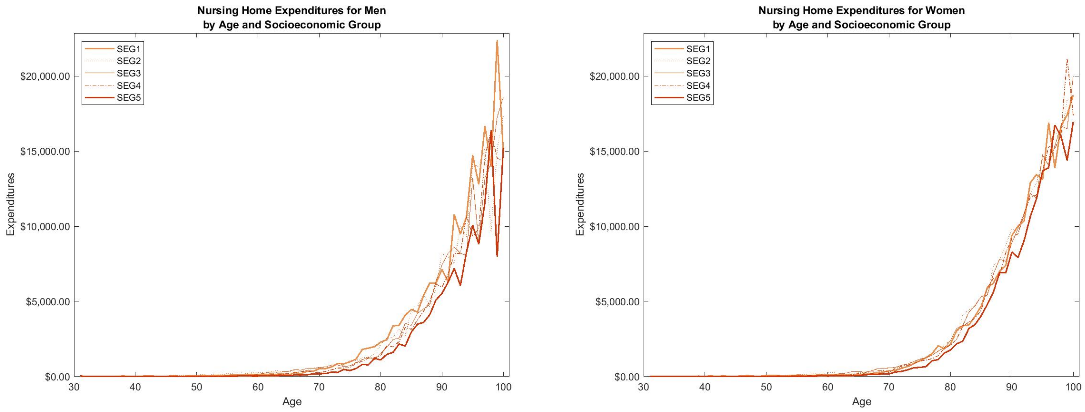

Figure A.9: Expenditures to nursing homes for males (left) and females (right) across socioeconomic groups in 2012.

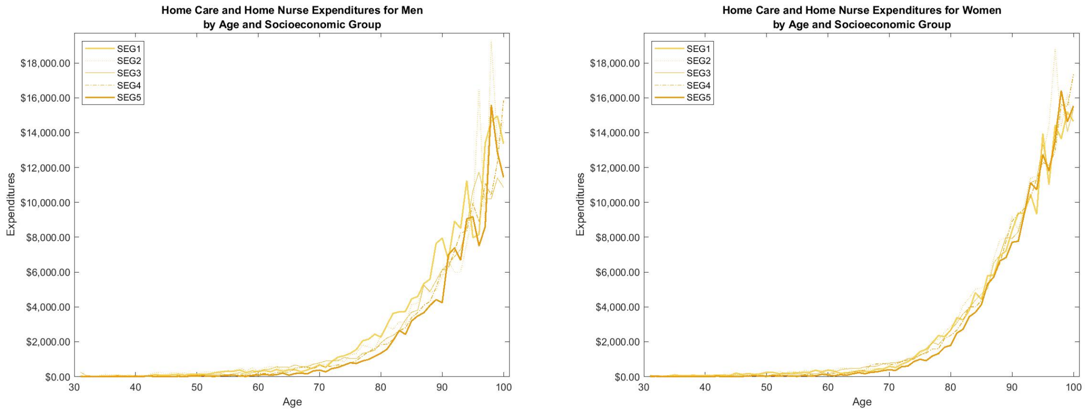

Figure A.10: Expenditures to home care and home nurses for males (left) and females (right) across socioeconomic groups in 2012.

B.2 Test of Equal Lifetime Healthcare Expenditures

Table A.2 tests the equality of lifetime healthcare expenditures across socioeconomic groups for various healthcare expenditure components. Tests for males are on lower diagonal elements and tests for females on the upper diagonal with greyscale background. We apply the test to two settings; the left-most part of the table where mortality only varies by gender and not socioeconomic groups, and, the right-most part of the table, where mortality vary by both gender and socioeconomic group. The right part correspond to our main results.

Table A.2: P-values from Welch Test of Equal Lifetime Healthcare Expenditures

P-values from Welch Test

Mortality by Gender

	SEG1	SEG2	SEG3	SEG4	SEG5	SEG1	SEG2	SEG3	SEG4	SEG5
	Inpatient Hospital					Outpatient Hospital				
SEG1		0.000	0.000	0.000	0.000		0.001	0.000	0.000	0.000
SEG2	0.003		0.000	0.000	0.000	0.539		0.000	0.000	0.000
SEG3	0.000	0.000		0.00001	0.00002	0.00005	0.000		0.043	0.399
SEG4	0.000	0.000	0.001		0.869	0.0003	0.00001	0.611		0.250
SEG5	0.000	0.000	0.009	0.399		0.205	0.053	0.004	0.019	
	Nursing Home					Home Care + Home Nurse				
SEG1		0.0001	0.000	0.000	0.000		0.00002	0.000	0.000	0.00003
SEG2	0.000		0.0002	0.003	0.074	0.00001		0.553	0.816	0.869
SEG3	0.000	0.033		0.469	0.048	0.000	0.256		0.713	0.441
SEG4	0.000	0.004	0.422		0.214	0.00002	0.797	0.416		0.685
SEG5	0.000	0.107	0.623	0.202		0.00005	0.929	0.332	0.875	
	Prescription Drugs					Primary Care Physician				
SEG1		0.000	0.000	0.000	0.000		0.378	0.605	0.775	0.000
SEG2	0.010		0.000	0.000	0.000	0.000		0.702	0.527	0.000
SEG3	0.000	0.000		0.000	0.000	0.000	0.0004		0.805	0.000
SEG4	0.000	0.000	0.0001		0.0001	0.000	0.000	0.000		0.000
SEG5	0.000	0.000	0.043	0.049		0.000	0.000	0.000	0.000	
	Total									
SEG1		0.889	0.00003	0.000	0.000					
SEG2	0.031		0.00005	0.000	0.000					
SEG3	0.0001	0.000		0.003	0.001					
SEG4	0.000	0.000	0.245		0.816					
SEG5	0.00004	0.000	0.887	0.312						

Mortality by Gender and Socioeconomic Group

	SEG1	SEG2	SEG3	SEG4	SEG5	SEG1	SEG2	SEG3	SEG4	SEG5
	Inpatient Hospital					Outpatient Hospital				
SEG1		0.000	0.000	0.000	0.000		0.000	0.000	0.000	0.000
SEG2	0.000		0.000	0.000	0.000	0.00001		0.000	0.000	0.000
SEG3	0.000	0.000		0.000	0.000	0.000	0.000		0.00001	0.000
SEG4	0.000	0.000	0.000		0.00001	0.000	0.000	0.000		0.020
SEG5	0.000	0.000	0.000	0.000		0.000	0.000	0.000	0.001	
	Nursing Home					Home Care + Home Nurse				
SEG1		0.000	0.0003	0.051	0.000		0.000	0.876	0.00001	0.000
SEG2	0.924		0.158	0.000	0.000	0.305		0.000	0.000	0.000
SEG3	0.000	0.000		0.000	0.000	0.000	0.000		0.00002	0.000
SEG4	0.000	0.000	0.000		0.000	0.000	0.000	0.000		0.000
SEG5	0.000	0.000	0.000	0.000		0.000	0.000	0.000	0.000	
	Prescription Drugs					Primary Care Physician				
SEG1		0.000	0.000	0.000	0.000		0.000	0.000	0.000	0.000
SEG2	0.000		0.000	0.000	0.000	0.004		0.000	0.000	0.000
SEG3	0.000	0.000		0.000	0.000	0.000	0.000		0.000	0.000
SEG4	0.000	0.000	0.000		0.000	0.000	0.000	0.000		0.049
SEG5	0.000	0.000	0.000	0.000		0.000	0.000	0.000	0.150	
	Total									
SEG1		0.0001	0.000	0.000	0.000					
SEG2	0.000		0.000	0.000	0.000					
SEG3	0.000	0.000		0.000	0.000					
SEG4	0.000	0.000	0.000		0.000					
SEG5	0.000	0.000	0.000	0.000						

Tests of equal lifetime healthcare expenditures by gender and socioeconomic group using the test statistic in equation (7) in the paper. When mortality vary only by gender, the variance in the denominator is $Var\left(\mathrm{LHE}_{s_1}-\mathrm{LHE}_{s_2}\right)=Var\left(\mathrm{LHE}_{s_1}\right)+Var\left(\mathrm{LHE}_{s_2}\right)-2\mathrm{Cov}\left(\mathrm{LHE}_{s_1},\mathrm{LHE}_{s_2}\right)$. The tests use bootstrapped standard errors (Efron, 1979).
Male test results are in lower diagonal elements, and female results are in upper diagonal elements of grey backgroundcolor.

C Robustness Estimates

C.1 Robustness: Lifetime Expenditures and Age 85 as Highest Age

Figure A.11 displays lifetime healthcare expenditures, with the maximum age capped at 85 instead of the top-coded age of 100 used in our main results. The estimates show a gradient compared to our main results in Figure 4 of the paper. Therefore, mortality differences and healthcare consumption patterns from age 85 to 100 have significant implications for estimates of lifetime healthcare expenditures. In particular, mortality and healthcare consumption patterns differ across socioeconomic groups from age 85 to 100. Due to our educational measure having age 90 as the maximum age, we prefer our affluence-based socioeconomic measure. Asaria et al. (2016) top-codes mortality and hospitalization rates at age 85+ and thus does not capture any differences in hospital expenditures from age 85 to 100. Table A.3 shows lifetime expenditures by gender, socioeconomic group, and cost component when we top-code our data at age 85, whereas Table A.4 provides p-values from the Welch test.

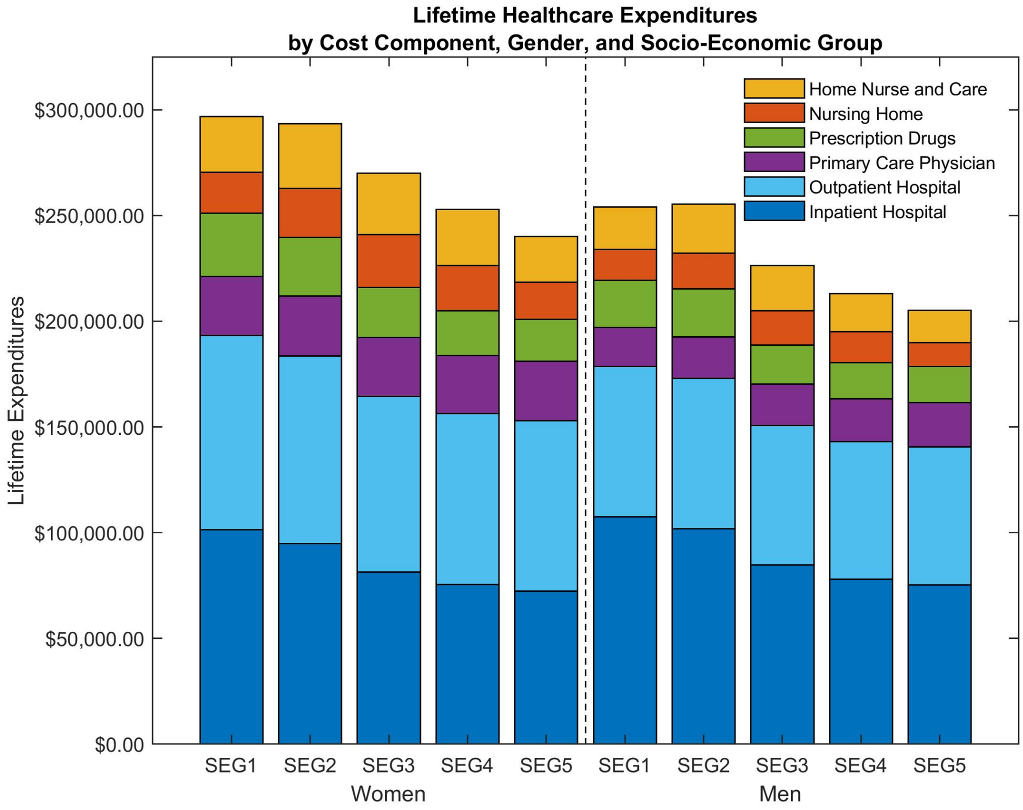

Figure A.11: Lifetime healthcare expenditures for females (left) and males (right) in socioeconomic groups top-coded at age 85+.

Table A.3: Lifetime healthcare expenditures by age, gender, socioeconomic group, and gender top-coded at age 85+.

Lifetime Healthcare Expenditures by Cost Component										
	Males					Females				
	SEG1	SEG2	SEG3	SEG4	SEG5	SEG1	SEG2	SEG3	SEG4	SEG5
Inpatient Hospital	107,526	101,797	84,725	77,960	75,282	101,497	94,864	81,305	75,520	72,440
	(953)	(926)	(791)	(825)	(760)	(819)	(795)	(652)	(692)	(845)
Outpatient Hospital	71,164	71,181	65,905	65,102	65,328	91,652	88,737	83,175	80,788	80,391
	(559)	(575)	(539)	(544)	(538)	(614)	(609)	(589)	(582)	(559)
Nursing Home	14,593	16,941	16,312	14,492	11,389	19,275	23,166	24,869	21,466	17,559
	(341)	(345)	(357)	(353)	(316)	(376)	(428)	(440)	(394)	(364)
Home Care + Home Nurse	19,908	23,143	21,395	18,061	15,252	26,477	30,579	29,166	26,499	21,684
	(420)	(434)	(465)	(444)	(442)	(481)	(528)	(517)	(530)	(479)
Prescription Drugs	22,431	22,833	18,293	17,169	17,039	29,825	27,790	23,716	21,007	19,791
	(143)	(143)	(128)	(121)	(113)	(151)	(144)	(132)	(113)	(103)
Primary Care Physician	18,270	19,490	19,703	20,265	20,904	28,062	28,201	27,808	27,563	28,183
	(91)	(95)	(86)	(85)	(83)	(108)	(102)	(95)	(89)	(90)
Total	253,892	255,384	226,334	213,049	205,193	296,788	293,338	270,040	252,843	240,048
	(1,647)	(1,625)	(1,472)	(1,409)	(1,330)	(1,635)	(1,648)	(1,505)	(1,382)	(1,353)

Amounts are in 2012 USD.

Table A.4: P-vales from Welch test of equal lifetime healthcare expenditures across socioeconomic groups using the bootstrapped standard errors. Male test results are in lower diagonal elements and female results in upper diagonal elements.

**P-values from Welch Test**										
	SEG1	SEG2	SEG3	SEG4	SEG5	SEG1	SEG2	SEG3	SEG4	SEG5
	Inpatient Hospital					Outpatient Hospital				
SEG1		0.000	0.000	0.000	0.000		0.001	0.000	0.000	0.000
SEG2	0.00002		0.000	0.000	0.000	0.983		0.000	0.000	0.000
SEG3	0.000	0.000		0.000	0.000	0.000	0.000		0.004	0.001
SEG4	0.000	0.000	0.000		0.005	0.000	0.000	0.394		0.623
SEG5	0.000	0.000	0.000	0.017		0.000	0.000	0.449	0.767	
	Nursing Home					Home Care + Home Nurse				
SEG1		0.000	0.000	0.0001	0.001		0.000	0.0001	0.976	0.000
SEG2	0.000		0.006	0.003	0.000	0.000		0.056	0.000	0.000
SEG3	0.0005	0.205		0.000	0.000	0.018	0.006		0.0003	0.000
SEG4	0.838	0.000	0.0003		0.000	0.003	0.000	0.000		0.000
SEG5	0.000	0.000	0.000	0.000		0.000	0.000	0.000	0.00001	
	Prescription Drugs					Primary Care Physician				
SEG1		0.000	0.000	0.000	0.000		0.350	0.078	0.0004	0.390
SEG2	0.048		0.000	0.000	0.000	0.000		0.005	0.000	0.895
SEG3	0.000	0.000		0.000	0.000	0.000	0.097		0.060	0.004
SEG4	0.000	0.000	0.000		0.000	0.000	0.000	0.000		0.000
SEG5	0.000	0.000	0.000	0.043		0.000	0.000	0.000	0.000	
	Total									
SEG1		0.137	0.000	0.000	0.000					
SEG2	0.519		0.000	0.000	0.000					
SEG3	0.000	0.000		0.000	0.000					
SEG4	0.000	0.000	0.000		0.000					
SEG5	0.000	0.000	0.000	0.00005						

C.2 Robustness: Lifetime Healthcare Expenditures Using Various Measures of Average Annual Healthcare Expenditures

C.2.1 Robustness: Lifetime Healthcare Expenditures Using 3-year Age Bands

Figure A.12 illustrates lifetime healthcare expenditures, where the mean of healthcare expenditures and mortality is calculated in three-year age bands. These results are almost identical to our main results in Figure 4. Table A.5 shows the corresponding lifetime expenditures by gender, socioeconomic group, and cost component, and Table A.6 provides p-values from the Welch test.

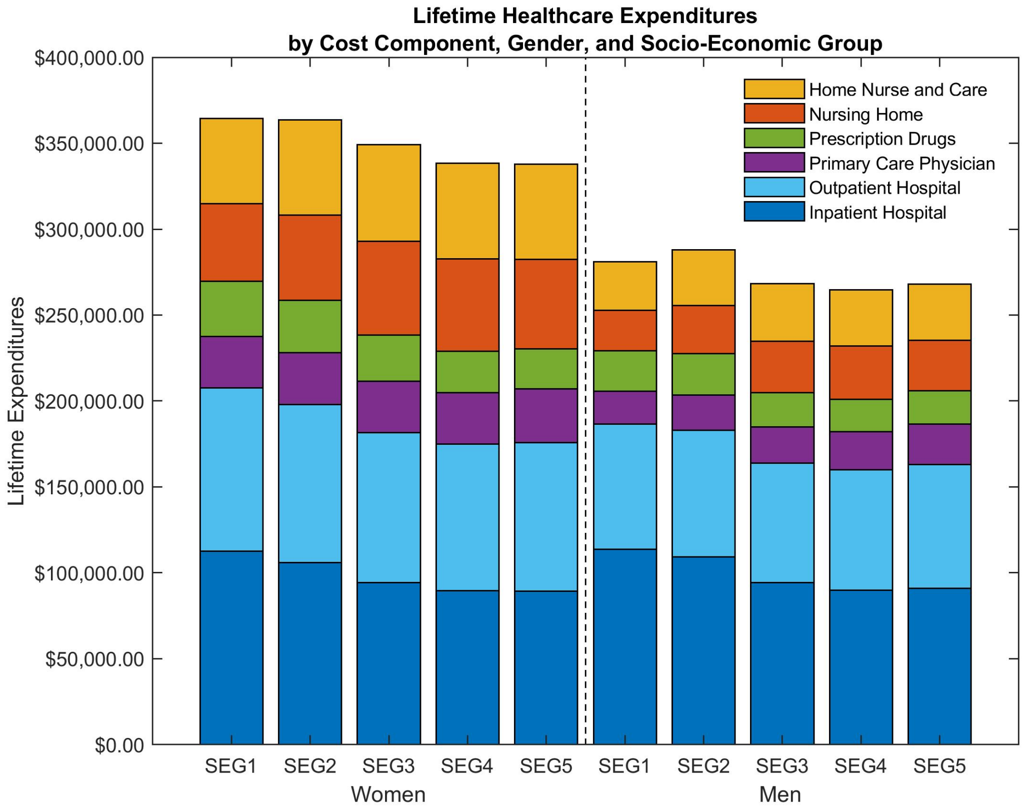

Figure A.12: Lifetime healthcare expenditures for females (left) and males (right) by socioeconomic group

Table A.5: Lifetime healthcare expenditures sex, socioeconomic group, and cost component

Lifetime Healthcare Expenditures by Cost Component										
	Males					Females				
	SEG1	SEG2	SEG3	SEG4	SEG5	SEG1	SEG2	SEG3	SEG4	SEG5
Inpatient Hospital	113,581	109,383	94,332	90,006	91,090	112,511	105,945	94,371	89,503	89,333
	(1,007)	(1,025)	(892)	(910)	(889)	(900)	(844)	(742)	(819)	(905)
Outpatient Hospital	73,085	73,634	69,479	69,915	71,969	95,023	92,064	87,206	85,483	86,487
	(645)	(603)	(608)	(606)	(623)	(639)	(578)	(601)	(599)	(618)
Nursing Home	23,415	27,836	29,908	30,793	29,416	44,963	49,780	54,573	53,591	52,062
	(573)	(655)	(719)	(767)	(723)	(847)	(892)	(930)	(922)	(887)
Home Care + Home Nurse	28,352	32,511	33,675	32,837	32,638	49,561	55,392	56,241	55,701	55,203
	(651)	(703)	(769)	(809)	(817)	(916)	(1,021)	(970)	(975)	(1,000)
Prescription Drugs	23,546	24,141	19,930	19,104	19,514	32,379	30,499	26,763	24,189	23,417
	(163)	(161)	(147)	(145)	(147)	(179)	(169)	(159)	(149)	(137)
Primary Care Physician	19,130	20,539	21,097	22,083	23,441	29,864	30,017	29,951	29,907	31,219
	(107)	(112)	(110)	(117)	(126)	(126)	(118)	(116)	(114)	(118)
Total	281,110	288,044	268,420	264,736	268,069	364,301	363,698	349,105	338,373	337,721
	(2,189)	(2,292)	(2,313)	(2,363)	(2,356)	(2,541)	(2,548)	(2,546)	(2,507)	(2,531)

Amounts are in 2012 USD.

Table A.6: P-vales from Welch test of equal lifetime healthcare expenditures across socioeconomic groups using the bootstrapped standard errors. Male test results are in lower diagonal elements and female results in upper diagonal elements.

**P-values from Welch Test**										
	SEG1	SEG2	SEG3	SEG4	SEG5	SEG1	SEG2	SEG3	SEG4	SEG5
	Inpatient Hospital					Outpatient Hospital				
SEG1		0.000	0.000	0.000	0.000		0.001	0.000	0.000	0.000
SEG2	0.003		0.000	0.000	0.000	0.534		0.000	0.000	0.000
SEG3	0.000	0.000		0.00001	0.00002	0.00005	0.000		0.042	0.404
SEG4	0.000	0.000	0.001		0.889	0.0003	0.00001	0.612		0.243
SEG5	0.000	0.000	0.010	0.394		0.213	0.055	0.004	0.018	
	Nursing Home					Home Care + Home Nurse				
SEG1		0.0001	0.000	0.000	0.000		0.00002	0.000	0.000	0.00003
SEG2	0.000		0.0002	0.003	0.070	0.00001		0.547	0.827	0.895
SEG3	0.000	0.033		0.453	0.051	0.000	0.264		0.695	0.456
SEG4	0.000	0.003	0.400		0.232	0.00002	0.761	0.453		0.722
SEG5	0.000	0.105	0.630	0.192		0.00004	0.907	0.355	0.863	
	Prescription Drugs					Primary Care Physician				
SEG1		0.000	0.000	0.000	0.000		0.376	0.615	0.801	0.000
SEG2	0.009		0.000	0.000	0.000	0.000		0.687	0.502	0.000
SEG3	0.000	0.000		0.000	0.000	0.000	0.0004		0.789	0.000
SEG4	0.000	0.000	0.0001		0.0001	0.000	0.000	0.000		0.000
SEG5	0.000	0.000	0.046	0.047		0.000	0.000	0.000	0.000	
	Total									
SEG1		0.867	0.00002	0.000	0.000					
SEG2	0.029		0.00005	0.000	0.000					
SEG3	0.0001	0.000		0.003	0.002					
SEG4	0.000	0.000	0.265		0.855					
SEG5	0.00005	0.000	0.915	0.318						

C.2.2 Robustness: Lifetime Healthcare Expenditures Using 5-year Age Bands

Figure A.13 depicts lifetime healthcare expenditures, with the mean of healthcare expenditures and mortality calculated in five-year age bands. These results are almost identical to our main results in Figure 4. Table A.7 shows the corresponding lifetime expenditures by gender, socioeconomic group, and cost component, and Table A.8 provides p-values from the Welch test.

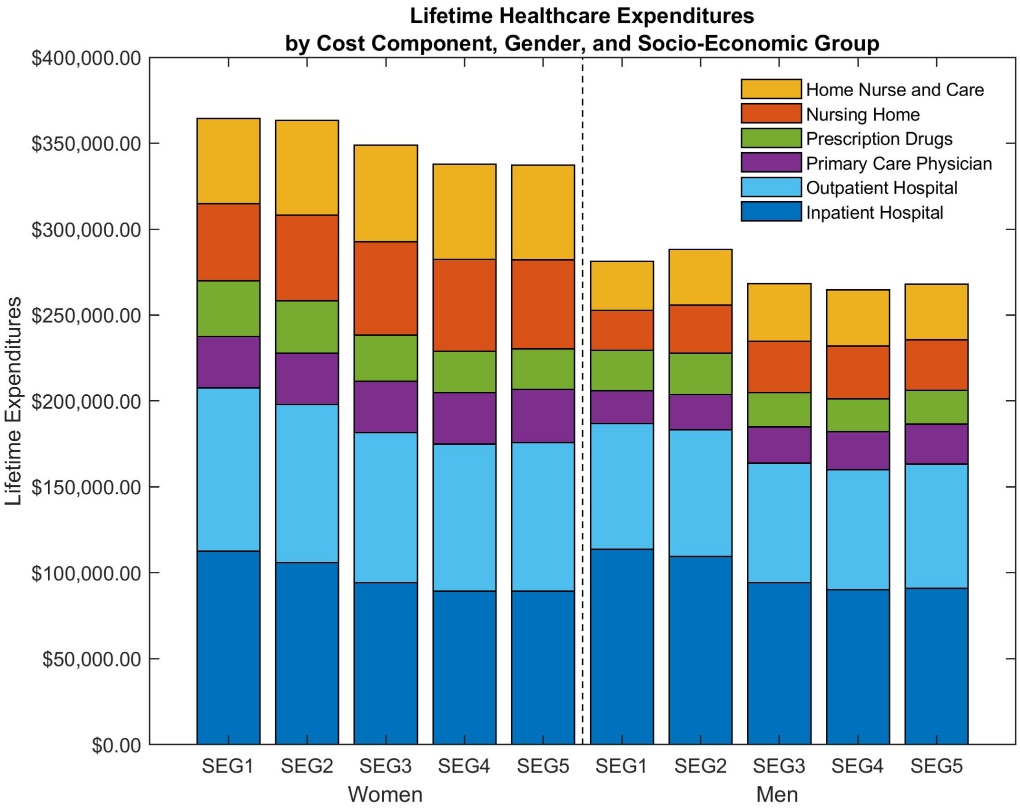

Figure A.13: Lifetime healthcare expenditures for females (left) and males (right) by socioeconomic group

Table A.7: Lifetime healthcare expenditures sex, socioeconomic group, and cost component

Lifetime Healthcare Expenditures by Cost Component										
	Males					Females				
	SEG1	SEG2	SEG3	SEG4	SEG5	SEG1	SEG2	SEG3	SEG4	SEG5
Inpatient Hospital	113,618	109,498	94,379	90,047	91,135	112,570	105,834	94,338	89,426	89,244
	(1,004)	(1,027)	(893)	(910)	(889)	(900)	(840)	(741)	(818)	(904)
Outpatient Hospital	73,178	73,698	69,540	69,956	72,055	95,110	92,036	87,197	85,468	86,464
	(645)	(604)	(610)	(608)	(625)	(639)	(578)	(601)	(600)	(619)
Nursing Home	23,358	27,785	29,813	30,716	29,364	44,883	49,642	54,492	53,403	51,927
	(568)	(648)	(709)	(757)	(714)	(840)	(879)	(924)	(914)	(881)
Home Care + Home Nurse	28,310	32,479	33,589	32,778	32,584	49,503	55,191	56,140	55,559	55,090
	(647)	(696)	(763)	(800)	(810)	(909)	(999)	(963)	(967)	(994)
Prescription Drugs	23,569	24,165	19,939	19,118	19,533	32,411	30,500	26,767	24,183	23,407
	(163)	(161)	(147)	(145)	(148)	(179)	(168)	(160)	(149)	(137)
Primary Care Physician	19,149	20,559	21,110	22,099	23,461	29,885	30,025	29,956	29,907	31,210
	(107)	(112)	(110)	(117)	(126)	(127)	(118)	(117)	(114)	(119)
Total	281,183	288,184	268,369	264,716	268,131	364,362	363,228	348,890	337,947	337,342
	(2,188)	(2,288)	(2,305)	(2,351)	(2,346)	(2,533)	(2,526)	(2,539)	(2,494)	(2,522)

Amounts are in 2012 USD.

Table A.8: P-vales from Welch test of equal lifetime healthcare expenditures across socioeconomic groups using the bootstrapped standard errors. Male test results are in lower diagonal elements and female results in upper diagonal elements.

P-values from Welch Test										
	SEG1	SEG2	SEG3	SEG4	SEG5	SEG1	SEG2	SEG3	SEG4	SEG5
	Inpatient Hospital					Outpatient Hospital				
SEG1		0.000	0.000	0.000	0.000		0.0004	0.000	0.000	0.000
SEG2	0.004		0.000	0.000	0.000	0.556		0.000	0.000	0.000
SEG3	0.000	0.000		0.00001	0.00001	0.00004	0.000		0.042	0.396
SEG4	0.000	0.000	0.001		0.881	0.0003	0.00001	0.629		0.248
SEG5	0.000	0.000	0.010	0.393		0.211	0.059	0.004	0.016	
	Nursing Home					Home Care + Home Nurse				
SEG1		0.0001	0.000	0.000	0.000		0.00003	0.000	0.00001	0.00003
SEG2	0.000		0.0001	0.003	0.066	0.00001		0.494	0.791	0.943
SEG3	0.000	0.035		0.402	0.044	0.000	0.283		0.670	0.448
SEG4	0.000	0.003	0.383		0.245	0.00001	0.778	0.464		0.736
SEG5	0.000	0.102	0.656	0.194		0.00004	0.922	0.366	0.864	
	Prescription Drugs					Primary Care Physician				
SEG1		0.000	0.000	0.000	0.000		0.416	0.681	0.895	0.000
SEG2	0.009		0.000	0.000	0.000	0.000		0.674	0.472	0.000
SEG3	0.000	0.000		0.000	0.000	0.000	0.0005		0.767	0.000
SEG4	0.000	0.000	0.0001		0.0001	0.000	0.000	0.000		0.000
SEG5	0.000	0.000	0.051	0.046		0.000	0.000	0.000	0.000	
	Total									
SEG1		0.751	0.00002	0.000	0.000					
SEG2	0.027		0.0001	0.000	0.000					
SEG3	0.0001	0.000		0.002	0.001					
SEG4	0.000	0.000	0.267		0.865					
SEG5	0.00005	0.000	0.942	0.304						

C.2.3 Robustness: Lifetime Healthcare Expenditures Using Smoothing-Splines

Figure A.14 presents lifetime healthcare expenditures, with the mean of healthcare expenditures and mortality calculated using smoothing splines. Specifically, we use the `smooth.spline` function from the `stats` package in R. These results are almost identical to our main results in Figure 4. Table A.9 shows the corresponding lifetime expenditures by gender, socioeconomic group, and cost component, and Table A.10 provides p-values from the Welch test.

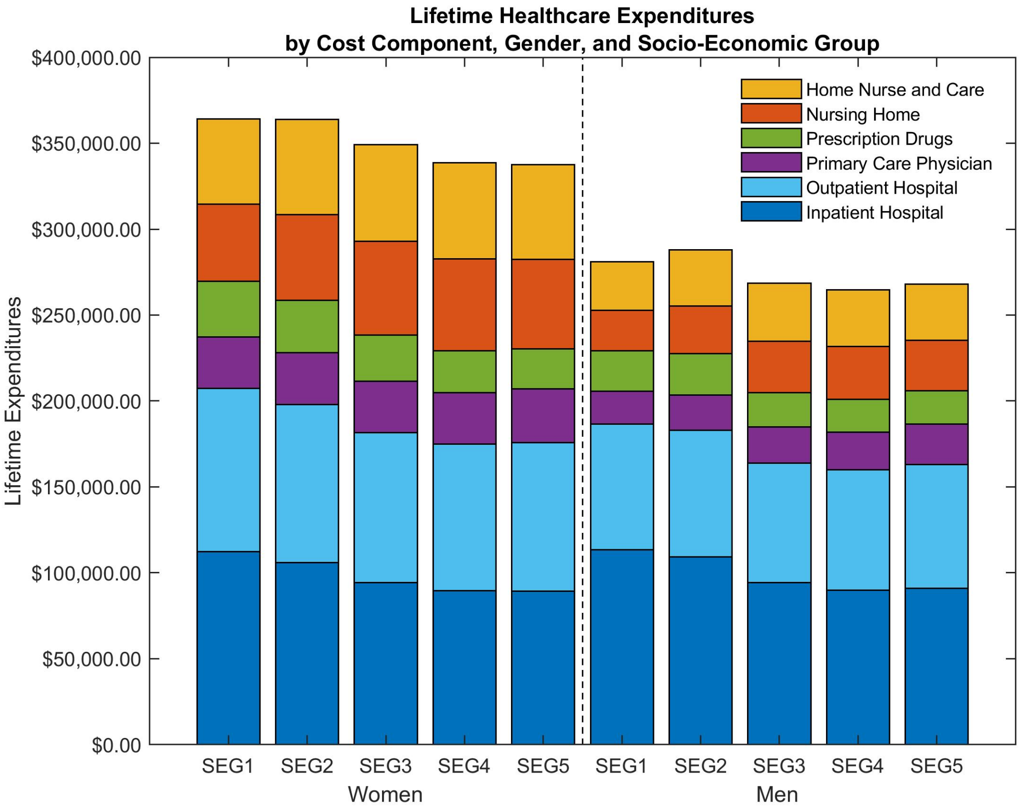

Figure A.14: Lifetime healthcare expenditures for females (left) and males (right) by socioeconomic group

Table A.9: Lifetime healthcare expenditures sex, socioeconomic group, and cost component

Lifetime Healthcare Expenditures by Cost Component										
	Males					Females				
	SEG1	SEG2	SEG3	SEG4	SEG5	SEG1	SEG2	SEG3	SEG4	SEG5
Inpatient Hospital	113,521	109,263	94,322	89,961	91,084	112,450	105,954	94,376	89,536	89,339
	(1,003)	(1,021)	(893)	(909)	(893)	(902)	(846)	(742)	(818)	(904)
Outpatient Hospital	73,036	73,592	69,471	69,895	71,934	94,983	92,059	87,190	85,491	86,490
	(643)	(602)	(609)	(606)	(622)	(641)	(579)	(601)	(599)	(618)
Nursing Home	23,422	27,833	29,921	30,800	29,403	44,985	49,804	54,561	53,665	52,030
	(576)	(659)	(727)	(776)	(727)	(851)	(900)	(933)	(928)	(890)
Home Care + Home Nurse	28,363	32,478	33,734	32,835	32,678	49,561	55,487	56,252	55,723	55,179
	(657)	(704)	(777)	(812)	(824)	(920)	(1,038)	(975)	(981)	(1,003)
Prescription Drugs	23,540	24,126	19,918	19,092	19,504	32,368	30,491	26,758	24,188	23,413
	(163)	(160)	(147)	(145)	(148)	(179)	(168)	(159)	(149)	(137)
Primary Care Physician	19,119	20,528	21,089	22,076	23,433	29,850	30,009	29,950	29,910	31,217
	(107)	(112)	(110)	(117)	(126)	(126)	(118)	(116)	(114)	(118)
Total	280,986	287,840	268,443	264,652	268,041	364,222	363,840	349,089	338,473	337,649
	(2,191)	(2,290)	(2,320)	(2,368)	(2,365)	(2,544)	(2,567)	(2,552)	(2,517)	(2,535)

Amounts are in 2012 USD.

Table A.10: P-vales from Welch test of equal lifetime healthcare expenditures across socioeconomic groups using the bootstrapped standard errors. Male test results are in lower diagonal elements and female results in upper diagonal elements.

**P-values from Welch Test**										
	SEG1	SEG2	SEG3	SEG4	SEG5	SEG1	SEG2	SEG3	SEG4	SEG5
	Inpatient Hospital					Outpatient Hospital				
SEG1		0.000	0.000	0.000	0.000		0.001	0.000	0.000	0.000
SEG2	0.003		0.000	0.000	0.000	0.529		0.000	0.000	0.000
SEG3	0.000	0.000		0.00001	0.00002	0.0001	0.000		0.045	0.417
SEG4	0.000	0.000	0.001		0.872	0.0004	0.00002	0.622		0.246
SEG5	0.000	0.000	0.010	0.378		0.218	0.056	0.005	0.019	
	Nursing Home					Home Care + Home Nurse				
SEG1		0.0001	0.000	0.000	0.000		0.00002	0.000	0.000	0.00004
SEG2	0.000		0.0002	0.003	0.079	0.00002		0.591	0.869	0.831
SEG3	0.000	0.033		0.496	0.050	0.000	0.231		0.702	0.443
SEG4	0.000	0.004	0.408		0.203	0.00002	0.740	0.424		0.698
SEG5	0.000	0.110	0.615	0.189		0.00004	0.854	0.351	0.892	
	Prescription Drugs					Primary Care Physician				
SEG1		0.000	0.000	0.000	0.000		0.357	0.559	0.722	0.000
SEG2	0.010		0.000	0.000	0.000	0.000		0.722	0.548	0.000
SEG3	0.000	0.000		0.000	0.000	0.000	0.0003		0.807	0.000
SEG4	0.000	0.000	0.0001		0.0001	0.000	0.000	0.000		0.000
SEG5	0.000	0.000	0.047	0.046		0.000	0.000	0.000	0.000	
	Total									
SEG1		0.916	0.00003	0.000	0.000					
SEG2	0.031		0.00005	0.000	0.000					
SEG3	0.0001	0.000		0.003	0.001					
SEG4	0.000	0.000	0.253		0.818					
SEG5	0.0001	0.000	0.903	0.311						

C.3 Robustness: Lifetime Expenditures by Education Group

Figure A.15 displays lifetime healthcare expenditures by the four educational groups defined in Appendix A.3. Data limits the upper age to 90. The least educated group, *basic*, spends approximately the same as the longest educated, *lhe*, which can also be seen in Table A.11. (Welch, 1947) test of equal lifetime healthcare expenditures across educational groups and by cost component in Table A.12 confirm that differences in lifetime healthcare expenditures are generally statistically insignificant. Just as for the affluence measure of socioeconomic status, the lowest socioeconomic groups spend more on inpatient hospital care and prescription drugs, as denoted by red in Table A.12. However, using educational groups reveals that females in the *basic* group (high school or

less) spend more on home care and home nurses than females with vocational/short higher education and females with medium higher education.

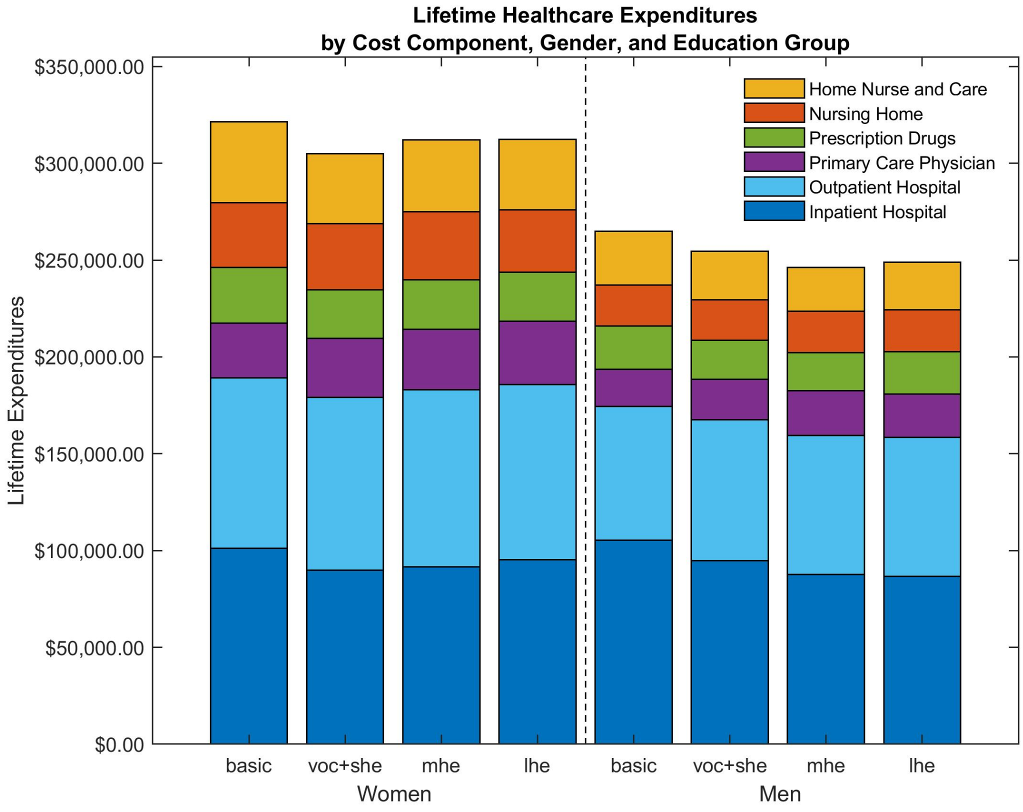

Figure A.15: Lifetime healthcare expenditures for females (left) and male (right) by education groups

Table A.11: Lifetime healthcare expenditures by age, gender, education group, and cost component

Lifetime Healthcare Expenditures by Cost Component								
	Males				Females			
	Basic	Voc.+Low	Medium	High	Basic	Voc.+Low	Medium	High
Inpatient Hospital	105,336	94,729	87,624	86,644	101,261	89,912	91,624	95,311
	(815)	(597)	(1,068)	(2,386)	(675)	(659)	(799)	(4,904)
Outpatient Hospital	69,065	72,748	71,790	71,695	87,929	89,304	91,513	90,333
	(443)	(425)	(880)	(1,657)	(459)	(541)	(621)	(2,698)
Nursing Home	21,158	21,082	21,399	21,545	33,507	34,319	35,172	32,102
	(283)	(350)	(666)	(1,476)	(316)	(569)	(800)	(3,743)
Home Care + Home Nurse	27,683	24,971	22,639	24,624	41,773	35,961	37,071	36,463
	(404)	(401)	(731)	(1,867)	(417)	(630)	(917)	(4,932)
Prescription Drugs	22,371	20,013	19,517	21,849	28,628	24,975	25,633	25,388
	(116)	(94)	(169)	(439)	(117)	(121)	(149)	(749)
Primary Care Physician	19,221	21,032	23,181	22,587	28,301	30,379	31,139	32,722
	(81)	(71)	(140)	(309)	(88)	(101)	(112)	(565)
Total	264,833	254,575	246,149	248,945	321,399	304,850	312,151	312,318
	(1,466)	(1,256)	(2,309)	(5,137)	(1,384)	(1,728)	(2,133)	(10,572)

Amounts are in 2012 USD.

Table A.12: P-vales from Welch test of equal lifetime healthcare expenditures across education groups using bootstrapped standard errors. Male test results are in lower diagonal elements and female results in upper diagonal elements.

**P-values from Welch Test**								
	Basic	Voc.+Low	Medium	High	Basic	Voc.+Low	Medium	High
	Inpatient Hospital				Outpatient Hospital			
Basic		0.000	0.000	0.227		0.053	0.000	0.380
Vocational+Low	0.000		0.098	0.275	0.000		0.007	0.709
Medium	0.000	0.000		0.458	0.006	0.327		0.670
High	0.000	0.001	0.708		0.125	0.538	0.960	
	Nursing Home				Home Care + Home Nurse			
Basic		0.212	0.053	0.708		0.000	0.000	0.283
Vocational+Low	0.867		0.385	0.558	0.000		0.319	0.920
Medium	0.738	0.674		0.423	0.000	0.005		0.904
High	0.796	0.760	0.928		0.109	0.856	0.322	
	Prescription Drugs				Primary Care Physician			
Basic		0.000	0.000	0.00002		0.000	0.000	0.000
Vocational+Low	0.000		0.001	0.586	0.904		0.000	0.000
Medium	0.000	0.010		0.748	0.000	0.004		0.000
High	0.251	0.00004	0.000		0.000	0.001	0.000	
	Total							
Basic		0.000	0.0003	0.394				
Vocational+Low	0.000		0.008	0.486				
Medium	0.000	0.001		0.988				
High	0.003	0.287	0.620					

References

Asaria, M., Doran, T., and Cookson, R. (2016). The costs of inequality: Whole-population modelling study of lifetime inpatient hospital costs in the English National Health Service by level of neighbourhood deprivation. *J Epidemiol Community Health*, 70(10):990–996. 5, 12

Bound, J., Geronimus, A. T., Rodriguez, J. M., and Waidmann, T. A. (2015). Measuring recent apparent declines in longevity: The role of increasing educational attainment. *Health Affairs*, 34(12):2167–2173. 5

Cairns, A. J. G., Kallestrup-Lamb, M., Rosenskjold, C., Blake, D., and Dowd, K. (2019). Modelling socio-economic differences in mortality using a new affluence index. *ASTIN Bulletin*, 49(3):555–590. 2, 4

Efron, B. (1979). Bootstrap methods: Another look at the jackknife. *The Annals of Statistics*, 7(1):1–26. 11

Jensen, V. M. and Rasmussen, A. W. (2011). Danish education registers. *Scandinavian Journal of Public Health*, 39(7_suppl):91–94. 4

Seshamani, M. and Gray, A. (2004). Ageing and health-care expenditure: The red herring argument revisited. *Health Economics*, 13(4):303–314. 7

UNESCO, I. f. S. (2012). *International standard classification of education: ISCED 2011*. UNESCO Institute for Statistics Montreal. 4

Welch, B. L. (1947). The generalization of student's' problem when several different population variances are involved. *Biometrika*, 34(1/2):28–35. 23
